# Pine-Extracted Phytosterol β-Sitosterol (APOPROSTAT^®^ Forte) Inhibits Both Human Prostate Smooth Muscle Contraction and Prostate Stromal Cell Growth, Without Cytotoxic Effects: A Mechanistic Link to Clinical Efficacy in LUTS/BPH

**DOI:** 10.3390/ph18121864

**Published:** 2025-12-06

**Authors:** Alexander Tamalunas, Felix Schierholz, Henrik Poth, Victor Vigodski, Michael Brandstetter, Anna Ciotkowska, Beata Rutz, Sheng Hu, Leo Federico Stadelmeier, Heiko Schulz, Stephan Ledderose, Nina Rogenhofer, Thomas Kolben, Christian Georg Stief, Martin Hennenberg

**Affiliations:** 1 Department of Urology, LMU University Hospital, LMU Munich, 81377 Munich, Germanymichael.brandstetter@med.uni-muenchen.de (M.B.);; 2 Department of Pathology, University Hospital Munich, LMU Munich, 81377 Munich, Germany; 3 Department of Obstetrics and Gynecology, University Hospital, LMU Munich, 81377 Munich, Germany

**Keywords:** lower urinary tract symptoms, phytotherapy, plant extracts, prostatic hyperplasia, smooth muscle contraction, phytosterols

## Abstract

**Introduction**: Medical treatment of lower urinary tract symptoms (LUTS) suggestive of benign prostatic hyperplasia (BPH) targets prostate smooth muscle tone for rapid relieve of symptoms and prostate size to prevent disease progression. Recently, EAU guidelines introduced phytomedicines for treatment of LUTS/BPH. Phytosterols may reduce the risk of prostate diseases and seem to be the smallest common denominator between different phytotherapeutic preparations. Thus, we investigated the effects of the highly concentrated phytosterol β-sitosterol on human prostate smooth muscle contraction and cellular functions, including contraction and growth of prostate stromal cells. **Materials and Methods**: APOPROSTAT^®^ forte capsules (>70% β-sitosterol, ethanol extract of *Pinus pinaster*) were dissolved in ethanol. Contractions were induced in human prostate tissues (*n* = 100) obtained from radical prostatectomy and assessed in organ bath setups. Cytoskeletal organization, proliferation, viability, cytotoxicity, and contraction in stromal cells (WPMY-1) were assessed using phalloidin staining, EdU, colony formation, CCK-8, flow cytometry, and matrix collagen assays. **Results**: APOPROSTAT^®^ forte (0.1–30 µg/mL) inhibited adrenergic, non-adrenergic, and neurogenic contractions of human prostate tissues by up to 71%, 69%, and 63%, respectively, in a dose-dependent manner. In WPMY-1 cells, it reduced proliferation and actin organization by up to 67% and 75% after 72 h, without affecting viability or inducing cytotoxicity. Colony formation decreased by up to 60% after 168 h, and contraction in collagen matrix assays was reduced by 57% in a concentration- and time-dependent manner. **Conclusions**: The natural phytosterol β-sitosterol effectively inhibits both prostate contraction and growth with a favorable safety profile, supporting its beneficial role in LUTS management through phytotherapy.

## 1. Introduction

Benign prostatic hyperplasia (BPH) refers to the non-malignant growth of prostate tissue, predominantly in the prostate’s transitional zone, and is a common cause of male lower urinary tract symptoms (mLUTS) [1]. Male LUTS refer to a symptom complex consisting of both voiding and storage disorders [2,3]. While obstructive voiding symptoms are most often a direct consequence of prostatic enlargement and exaggerated prostate smooth muscle tone, storage symptoms in men may occur both secondary to or independent of prostatic obstruction [2,4].

The prevalence of BPH increases with age from around half of the 50-year-old male population to >80% in men above the age of 80 years [1,5]. An unfavorable balance between stromal and epithelial cell proliferation rate and pro-apoptotic factors in the prostate’s transitional zone surrounding the urethra leads to concomitant compression of the male urethra, defined as benign prostatic obstruction (BPO) [5]. Although prostate size by itself may not be a definitive predictor of clinically relevant LUTS secondary to BPO, it increases with age, with studies indicating an annual growth of up to 2.5% [6]. In 2018, it was estimated that 612 million men worldwide were affected by LUTS/BPO, with annual costs peaking up to five billion USD for medical treatment [7]. Thus, LUTS/BPH add to the already heavy socioeconomic burden of further age-related diseases. With the ongoing demographic transition, and as age is a significant predictor of BPH and subsequent LUTS, the prevalence of mLUTS will rise dramatically within the next two decades. This is undermined by the estimation that, by the year 2040, 1 in 4 Americans will be above the age of 65 [6,8]. However, data today already show that BPH is the most common acute and chronic outpatient diagnosis in post-industrial societies where life expectancy steadily increases and birth rates plummet, with LUTS-associated symptoms making up more than half of all diagnoses [9].

Thus, adequate medical treatment is urgently warranted. Prostate smooth muscle contraction and stromal growth are the main pharmacologic targets in managing male lower urinary tract symptoms (mLUTS). Smooth muscle contraction is mediated by agonist-activated contractile receptors, whereas hyperplastic growth depends on growth factors and hormones such as dihydrotestosterone (DHT), produced from testosterone via 5α-reductase (5-AR) [4,10]. Standard treatment for LUTS suggestive of BPH targets both dynamic and static components: α_1_-adrenoceptor antagonists (α_1_-blockers) reduce smooth muscle contraction to improve urinary flow, while 5-AR inhibitors (5-ARIs) block testosterone conversion to DHT, decreasing prostate volume [2]. Alpha_1_-blockers typically improve IPSS and Q_max_ by up to 40%, while 5-ARIs can reduce prostate size by 18–28%. Common α_1_-blocker adverse effects include orthostatic hypotension, dizziness, falls, ejaculatory dysfunction, and intraoperative floppy iris syndrome [11]. 5-ARIs may cause endocrine-related side effects such as hot flushes, reduced libido, and persistent sexual or psychological symptoms known as post-finasteride syndrome [2,12,13].

Ongoing research in the field has prompted a shift among urologists away from the traditional reliance on α_1_-blocker monotherapy toward a new paradigm emphasizing combination pharmacotherapy. This evolution reflects the growing recognition of the multifactorial nature of male LUTS, particularly the increasing prevalence of mixed symptoms, involving both storage and voiding components [1,3]. Combination regimens aim to simultaneously target distinct pathophysiological mechanisms, i.e., smooth muscle contraction and cell growth. However, this therapeutic strategy is associated with an additive risk of adverse events inherent to either of the combined drug classes [2,4]. Consequently, discontinuation rates for these combination therapies can reach as high as 90% within one year after treatment initiation, primarily due to insufficient therapeutic efficacy in combination with intolerable adverse effects [12,14]. These limitations underscore the unmet medical need within current pharmacological approaches and may partly account for the growing patient-driven interest in over-the-counter (OTC) phytotherapeutic alternatives [12,14].

The European Association of Urology (EAU) has recently incorporated a recommendation for the use of hexane-extracted saw palmetto (*Serenoa repens*; HESr) in its guidelines for the management of non-neurogenic male LUTS, marking the first official endorsement of a specific phytotherapeutic agent for the treatment of LUTS secondary to BPH [2]. Although previously absent from formal clinical recommendations, phytotherapeutics have been among the most extensively studied treatment modalities for LUTS/BPH in randomized clinical trials, demonstrating clinically meaningful symptom relief in affected men [15,16]. The limited recommendation of phytomedicines was largely due to the heterogeneity of available preparations and a lack of robust clinical data demonstrating a reduction in disease progression or prevention of significant complications. Phytomedicines are now particularly recommended for patients seeking to avoid the treatment-related adverse events associated with conventional pharmacologic therapies. These plant-based preparations constitute a rapidly expanding segment of the therapeutic market, where there is currently an unmet medical need and growth is driven in part by patient preference for non-conventional therapies [17]. Most phytotherapeutics are available over the counter, obviating the need for specialist consultation, which may pose a logistical or mental barrier for some patients [18]. Recent preclinical studies have provided new insights into the potential mechanisms of action of HESr preparations, particularly Permixon^®^, offering for the first time a plausible scientific basis for its in vivo efficacy [19]. These findings suggest that phytomedicines may reduce smooth muscle tone in the hyperplastic prostate and simultaneously inhibit prostatic growth. However, what proportion of these effects can be attributed to the plant species they are extracted from, the extraction technique (hexane vs. ethanol vs. lipido-sterolic), or the active components remains uncertain, as data are often limited to non-urological or non-human tissues [20,21], or have not been reproducible in vivo [22]. While differences in manufacturing processes are difficult to assess, the main active components appear to be free fatty acids, lectins, and phytosterols, with the latter as the smallest common denominator of phytotherapeutic preparations used for LUTS/BPH [23,24,25,26]. Drugs derived from plants have a long tradition in Europe for the medical treatment of LUTS/BPH, although neither mechanisms of action nor a precise classification of the active compounds for many of these drugs has ever been reported. Thus, given its composition—particularly its high phytosterol content (>70% β-sitosterol)—and its convincing clinical data, APOPROSTAT^®^ forte is likely to exhibit in vitro mechanisms of action [27,28,29]. Compared to the clinical data derived from two systematic reviews focused on data from fifteen randomized controlled trials (RCTs) and as β-sitosterol is the main active component in HESr preparations [26], highly concentrated β-sitosterol used within the APOPROSTAT^®^ forte formulation seems highly efficacious [15,16]. Thus, to the best of our knowledge, there is currently no study explaining the in vivo efficacy of highly concentrated β-sitosterol in clinical trials [27,28,29]. Therefore, we examined the effects of APOPROSTAT^®^ forte with the highest phytosterol content (>70% β-sitosterol) on the German market on human prostate and contraction and on cellular functions of human prostate stromal cells.

## 2. Results

### 2.1. Contractility Measurements

**Figure 1:**
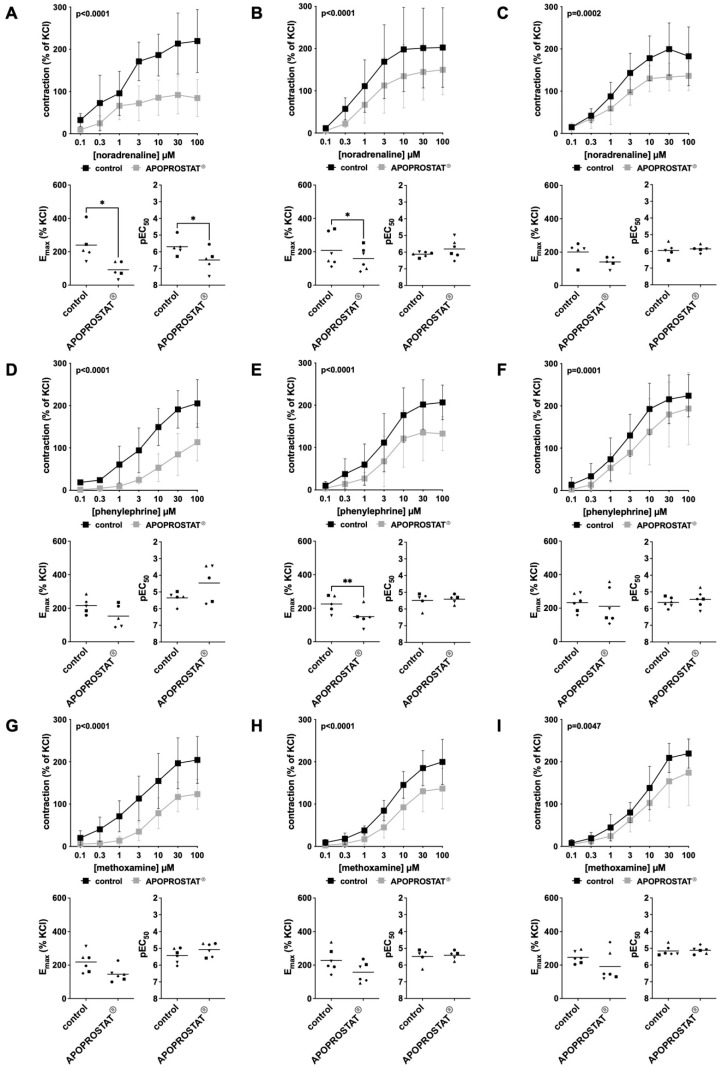
Effects of APOPROSTAT^®^ forte on adrenergic contractions of human prostate smooth muscle tissues. Adrenergic contractions were induced by subtype unselective α_1_-agonist noradrenaline (**A**–**C**), α_1A_- and α_1B_-selective agonist phenylephrine (**D**–**F**), and α_1A_-selective agonist methoxamine (**G**–**I**), after addition of APOPROSTAT^®^ forte (30, 10, and 3 µg/mL, respectively) or equal amounts of ethanol for controls. To eliminate heterogeneities due to individual variations or varying smooth muscle content, tensions were expressed as percentages (%) of contraction by high-molar KCl, being assessed before application of the investigative drug or ethanol. Each experiment used strips from different patients (*n* = 50), and data are graphed as means ± SD from n ≥ 5 different patients per individual series. Tissue from each patient was allocated to the control and drug group examined in the same experiment, resulting in paired groups and identical group sizes in each diagram. Overall *p*-values reflect comparison in two-way ANOVA between treatment and control groups (*p*-values for whole groups in inserts). All single E_max_ values (**left**) with pEC_50_ values (**right**) for agonist-induced contractions are shown in scatter plots beneath their corresponding concentration−response curves (* *p* < 0.05, ** *p* < 0.01).

**Figure 2:**
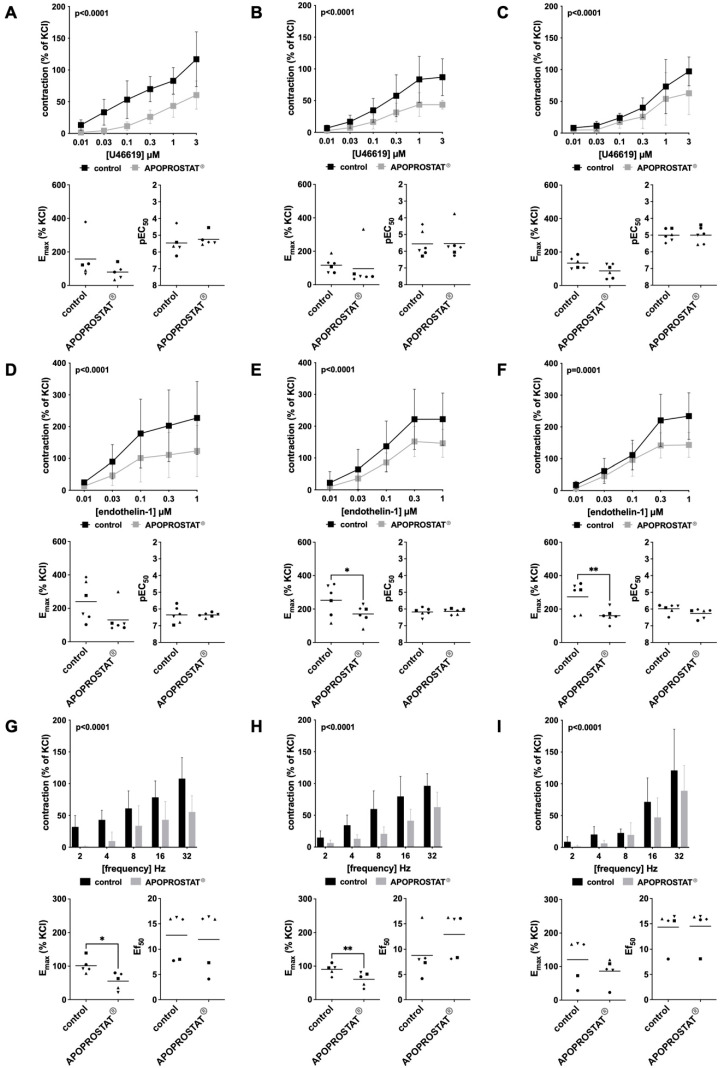
Effects of APOPROSTAT^®^ forte on non-adrenergic and neurogenic contractions of human prostate smooth muscle tissues. Non-adrenergic contractions were induced by thromboxane A_2_ analog U46619 (**A**–**C**) and endothelin-1 (**D**–**F**), and neurogenic contraction was induced by electric field stimulation (EFS) (**G**–**I**), after addition of APOPROSTAT^®^ forte (30, 10, and 3 µg/mL, respectively) or equal amounts of ethanol for controls. To eliminate heterogeneities due to individual variations or varying smooth muscle content, tensions were expressed as percentages (%) of contraction by high-molar KCl, being assessed before application of the investigative drug or ethanol. Each experiment used strips from different patients (*n* = 50), and data are graphed as means ± SD from *n* ≥ 5 different patients per individual series. Tissue from each patient was allocated to the control and drug group examined in the same experiment, resulting in paired groups and identical group sizes in each diagram. Overall *p*-values reflect the comparison in two-way ANOVA between treatment and control groups (*p*-values for whole groups in inserts). All single E_max_ values (**left**) with pEC_50_ values and Ef_50_ values (**right**) for agonist-induced and EFS-induced contractions, respectively, are shown in scatter plots beneath their corresponding concentration−response curves (* *p* < 0.05; ** *p* < 0.01).

Effect sizes become obvious from frequency and concentration response curves and from scatter plots given in Figure 1 and Figure 2. Detailed results are given in Supplementary File S1, in which the AUC represents an overview for comparison of concentration response and frequency response curves with APOPROSTAT^®^ forte to corresponding curves without APOPROSTAT^®^ forte. Results from post hoc analyses at each concentration in concentration−response curves, using Sidak’s multiple comparisons test, can be found in Supplementary Files S2–S4. While detailed results for E_max_, pEC_50_, and Ef_50_ can be found in Supplementary File S1, only statistically significant E_max_, pEC_50_ values for contractile agonists, and Ef_50_ values of the maximum EFS-induced contraction are stated in the following text.

#### 2.1.1. Effects of APOPROSTAT^®^ Forte on Adrenergic Contractions of Human Prostate Tissues

Human prostate tissue contraction was induced by the adrenergic agonists, noradrenaline, phenylephrine, and methoxamine, following incubation with APOPROSTAT^®^ forte at 30, 10, and 3 µg/mL. Noradrenaline-induced contractions were inhibited, and the AUC reduced in concentration response curves (0.1 to 100 µM) by around 55%, 34%, and 29% for concentrations of 30, 10, and 3 µg/mL APOPROSTAT^®^ forte, respectively (Figure 1A–C). E_max_ was reduced at concentrations of 30 and 10 µg/mL APOPROSTAT^®^ forte, amounting to 240 [114 to 366]% of KCl-induced contractions in controls and 92 [33 to 151]% of KCl-induced contractions after application of 30 µg/mL APOPROSTAT^®^ forte (MD 148 [52 to 244]%, *p* = 0.013, Figure 1A) and to 208 [105 to 312]% of KCl-induced contractions in controls and 159 [88 to 230]% of KCl-induced contractions after application of 10 µg/mL APOPROSTAT^®^ forte (MD 49 [3 to 95]%, *p* = 0.0422, Figure 1B). EC_50_ values decreased at concentrations of 30 µg/mL APOPROSTAT^®^ forte, reflected by increases of pEC_50_ values from 5.7 [5.0 to 6.4] for controls to 6.5 [5.6 to 7.4] for 30 µg/mL APOPROSTAT^®^ forte (MD 0.8 [0.1 to 1.5], *p* = 0.0356, Figure 1A).

Phenylephrine-induced contractions were inhibited and the AUC in concentration−response curves (0.1 to 100 µM) were reduced by around 63%, 38%, and 25% for concentrations of 30, 10, and 3 µg/mL APOPROSTAT^®^ forte, respectively (Figure 1D–F). E_max_ was reduced only at a concentration of 10 µg/mL APOPROSTAT^®^ forte, amounting to 226 [162 to 290]% of KCl-induced contractions in controls and 150 [76 to 223]% of KCl-induced contractions after application of 10 µg/mL APOPROSTAT^®^ forte (MD 76 [34 to 118]%, *p* = 0.0075, Figure 1E). EC_50_ values remained unchanged for phenylephrine-induced contractions.

Methoxamine-induced contractions were inhibited and the AUC in concentration−response curves (0.1 to 100 µM) were reduced by around 54%, 37%, and 26% for concentrations of 30, 10, and 3 µg/mL APOPROSTAT^®^ forte, respectively (Figure 1G–I). E_max_ and EC_50_ values remained unchanged for methoxamine-induced contractions.

#### 2.1.2. Effects of APOPROSTAT^®^ Forte on Non-Adrenergic Contractions of Human Prostate Tissues

Human prostate tissue contraction was induced by the non-adrenergic agonists thromboxane A_2_ analogue U46619 and endothelin-1 following incubation with APOPROSTAT^®^ forte at 30, 10, and 3 µg/mL. Contractions induced by U46619 were inhibited and the AUCs in concentration response curves (0.01 to 30 µM) were reduced by around 62%, 49%, and 33% for concentrations of 30, 10, and 3 µg/mL APOPROSTAT^®^ forte, respectively (Figure 2A–C). E_max_ and EC_50_ values remained unchanged for U46619-induced contractions.

Contractions induced by endothelin-1 were inhibited and the AUCs in concentration response curves (0.01 to 1.0 µM) were reduced by around 45%, 35%, and 31% for concentrations of 30, 10, and 3 µg/mL APOPROSTAT^®^ forte, respectively (Figure 2D–F). E_max_ was reduced at concentrations of 10 and 3 µg/mL APOPROSTAT^®^ forte, amounting to 253 [153 to 352]% of KCl-induced contractions in controls and 171 [116 to 226]% of KCl-induced contractions after application of 10 µg/mL APOPROSTAT^®^ forte (MD 82 [24 to 140]%, *p* = 0.0148, Figure 2E) and to 273 [181 to 366]% of KCl-induced contractions in controls and 161 [118 to 203]% of KCl-induced contractions after application of 3 µg/mL APOPROSTAT^®^ forte (MD 112 [43 to 182]%, *p* = 0.0086; Figure 2F). EC_50_ values remained unchanged for endothelin 1-induced contractions.

#### 2.1.3. Effects of APOPROSTAT^®^ Forte on Neurogenic Contractions of Human Prostate Tissues

Neurogenic contractions of human prostate tissue contractions were induced by electric field stimulation (EFS) following incubation with APOPROSTAT^®^ forte at 30, 10, and 3 µg/mL. EFS-induced contractions were reduced by around 55%, 52%, and 34% for concentrations of 30, 10, and 3 µg/mL APOPROSTAT^®^ forte, respectively (Figure 2G–I). E_max_ was reduced at concentrations of 30 and 10 µg/mL APOPROSTAT^®^ forte, amounting to 101 [72 to 130]% of KCl-induced contractions in controls and 55 [23 to 87]% of KCl-induced contractions after application of 30 µg/mL APOPROSTAT^®^ forte (MD 46 [12 to 77]%, *p* = 0.0195, Figure 2G) and to 90 [70 to 110]% of KCl-induced contractions in controls and 61 [35 to 87]% of KCl-induced contractions after application of 10 µg/mL APOPROSTAT^®^ forte (MD 29 [14 to 45]%, *p* = 0.0056; Figure 2H). Ef_50_ values remained unchanged for EFS-induced contractions.

#### 2.1.4. Effects of APOPROSTAT^®^ Forte on the Contractility of WPMY-1 Cells

**Figure 3:**
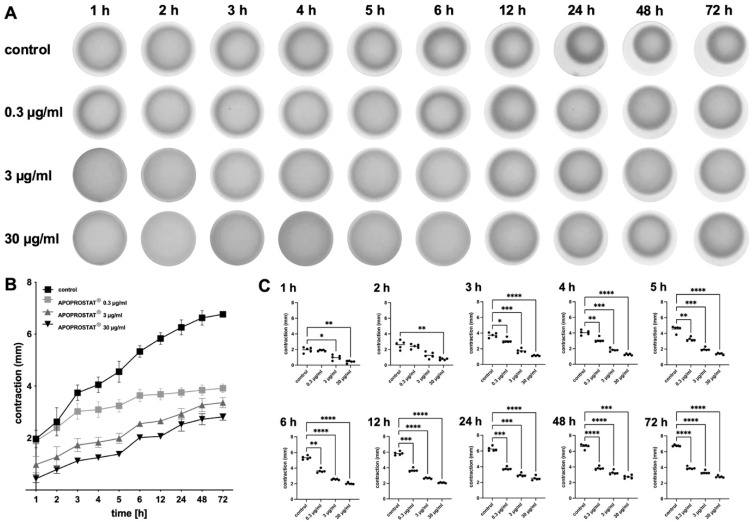
Effects of APOPROSTAT^®^ forte on contraction of WPMY-1 cells. Contraction of WPMY-1 cells over time was assessed by matrix contraction assays, starting after addition of APOPROSTAT^®^ forte (30, 3, and 0.3 µg/mL, respectively) or equal amounts of ethanol for controls. Contractions are expressed as decreases in matrix plug diameter during indicated periods. Shown are representative pictures for each condition (**A**), contractions over time (means with SD) (**B**), and single values from each sample and each single experiment for each time point in scatter plots (**C**), including *p*-values from two-way ANOVA with Tukey’s test (vs. control) in (**B**) and one-way ANOVA with Dunnett’s test in (**C**) (* *p* < 0.05; ** *p* < 0.01; *** *p* < 0.001; **** *p* < 0.0001).

Contractions of WPMY-1 cells were assessed 1 to 72 h after seeding to matrix plugs in contraction assays, where they increased over time (Figure 3). While detailed results for each concentration and time point can be taken from Supplementary Files S5 and S6, the AUCs are given following incubation with APOPROSTAT^®^ forte for concentrations of 30, 3, and 0.3 µg/mL, compared to volume equivalent amounts of ethanol for controls. After 72 h, the AUCs for controls amounted to 43.4 [42.0 to 44.7] and were reduced in a concentration-dependent manner: the AUC decreased to 15.5 [14.9 to 16.1], 20.4 [19.4 to 21.3], and 29.6 [28.6 to 30.5] for 30, 3, and 0.3 µg/mL APOPROSTAT^®^ forte, respectively. This corresponded to the concentration-dependent reduction in AUC by 64%, 53%, and 32% for concentrations of 30, 3, and 0.3 µg/mL APOPROSTAT^®^ forte, respectively (Figure 3A).

### 2.2. Cell Culture Studies

#### 2.2.1. Effects of APOPROSTAT^®^ Forte on Actin Organization of WPMY-1 Cells

**Figure 4:**
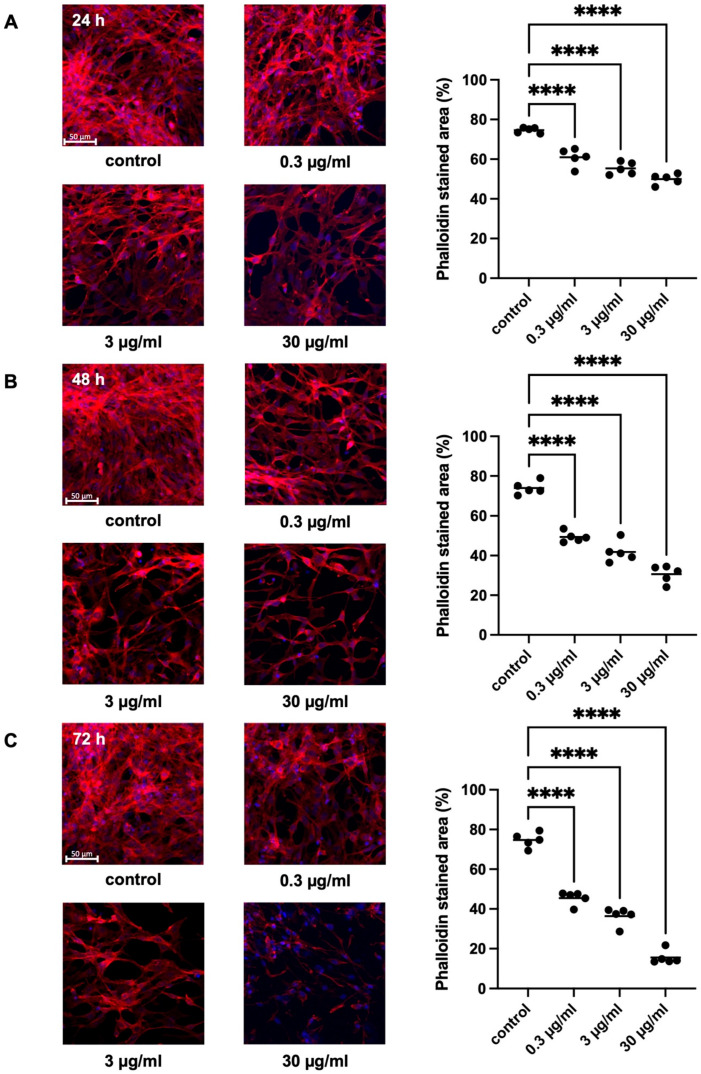
Inhibition of prostate stromal cell actin polymerization and organization by APOPROSTAT^®^ forte. WPMY-1 cells were incubated with different concentrations of APOPROSTAT^®^ forte, i.e., 0.3, 3, or 30 μg/mL, or equal amounts of solvent (ethanol) for controls. Shown are percentages of phalloidin-stained areas (single values from all experiments together with means) for each concentration after 24 h (**A**), 48 h (**B**), and 72 h (**C**), using cell cultures from *n* = 5 independent experiments for each concentration and time. The cells were either allocated to a control (ethanol) or drug (APOPROSTAT^®^ forte) group and incubated for 24, 48, and 72 h, respectively. Actin filaments were visualized by phalloidin staining, while the nuclei were visualized using DAPI staining. Shown are exemplary images of cell proliferation after 24, 48, and 72 h (**left**) and quantification of all experiments (**right**), including scale bars (50 µm) (**** *p* < 0.0001).

Phalloidin-stained actin filaments in ethanol-treated control cells were arranged to long bundles of thin protrusions, with elongations from adjacent cells overlapping each other (Figure 4). APOPROSTAT^®^ forte (0.3, 3, and 30 µg/mL) caused concentration- and time-dependent inhibition of actin filament polymerization, after incubation for 24, 48, and 72 h (Supplementary File S7). This resulted in a rounded cell shape without any protrusions (Figure 4A–C). After 24 h, APOPROSTAT^®^ forte caused respective regression of phalloidin-stained areas from 74.6 [72.9 to 76.3]% in solvent-treated control cells to 60.9 [55.4 to 66.5]%, 55.4 [51.5 to 59.2]%, and 50.0 [46.7 to 53.2] for incubation with 0.3, 3, and 30 µg/mL APOPROSTAT^®^ forte (*p* < 0.0001 for 0.3, 3, and 30 µg/mL APOPROSTAT^®^ forte vs. control, respectively; Figure 4A). After 48 h, APOPROSTAT^®^ forte caused respective regression of phalloidin-stained areas from 74.0 [69.9 to 78.0]% in solvent-treated control cells to 49.3 [46.1 to 52.6]%, 41.8 [35.3 to 48.3]%, and 30.7 [25.3 to 36.0]% for incubation with 0.3, 3, and 30 µg/mL APOPROSTAT^®^ forte (*p* < 0.0001 for 0.3, 3, and 30 µg/mL APOPROSTAT^®^ forte vs. control, respectively; Figure 4B). After 72 h, APOPROSTAT^®^ forte caused respective regression of phalloidin-stained areas from 74.7 [70.1 to 79.3]% in solvent-treated control cells to 45.5 [41.3 to 49.7]%, 36.3 [30.8 to 41.9]%, and to 15.5 [11.2 to 19.9]% for incubation with 0.3, 3, and 30 µg/mL APOPROSTAT^®^ forte (*p* < 0.0001 for 0.3, 3, and 30 µg/mL APOPROSTAT^®^ forte vs. control, respectively; Figure 4C). This corresponds to obvious and robust regression of phalloidin-stained areas, i.e., amounting to a relative decrease of the actin-stained area of 79% after exposure at the highest concentration of APOPROSTAT^®^ forte (30 µg/mL) for 72 h (Figure 4C).

#### 2.2.2. Effects of APOPROSTAT^®^ Forte on Proliferation of WPMY-1 Cells

**Figure 5:**
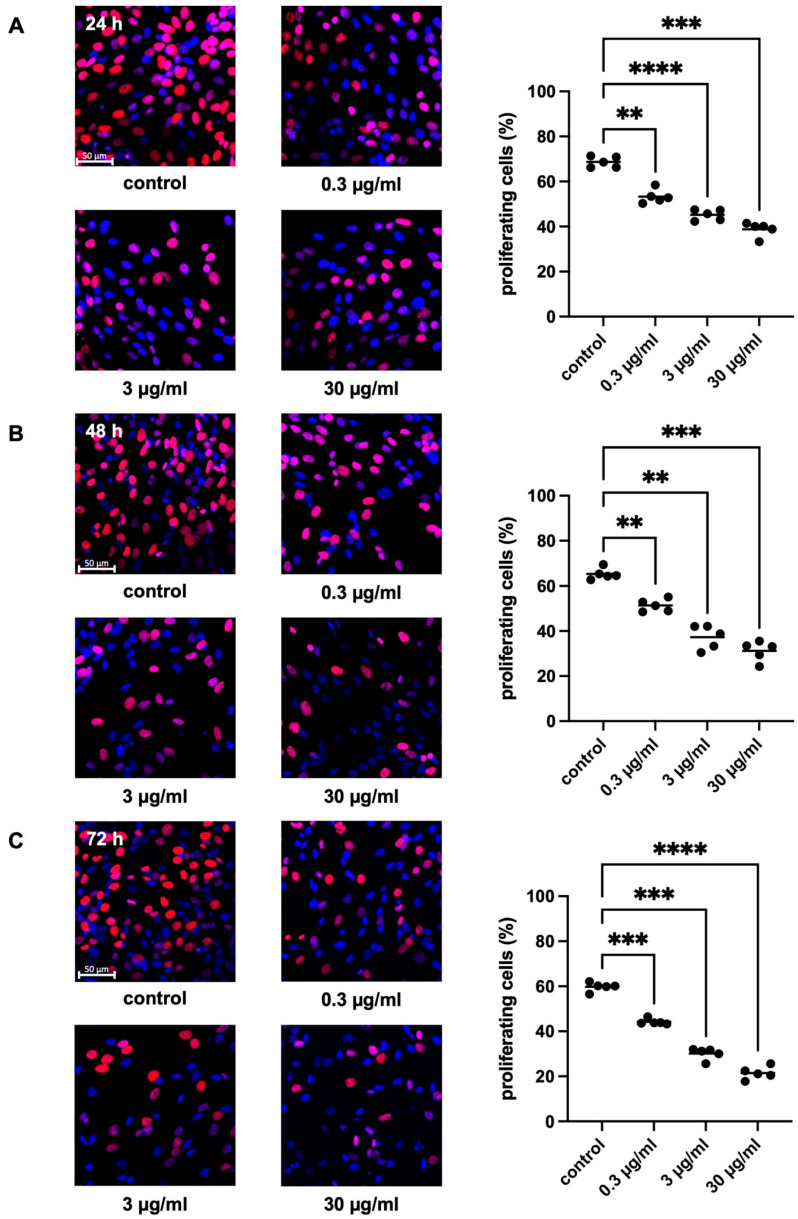
Inhibition of prostate stromal cell proliferation by APOPROSTAT^®^ forte. WPMY-1 cells were incubated with different concentrations of APOPROSTAT^®^ forte, i.e., 0.3, 3, or 30 μg/mL, or equal amounts of solvent (ethanol) for controls. Shown are percentages of proliferating cells (single values from each experiment together with means) for each concentration after 24 h (**A**), 48 h (**B**), and 72 h (**C**), using cell cultures from *n* = 5 independent experiments for each concentration and time. The cells were either allocated to a control (ethanol) or drug (APOPROSTAT^®^ forte) group and incubated for 24, 48, and 72 h. Proliferating cells were detected by EdU staining and counterstaining of all nuclei with DAPI, resulting in blue-colored nuclei for non-proliferating cells and red nuclei for proliferating cells. Shown are exemplary images of cell proliferation after 24, 48, and 72 h (**left**) and quantification of all experiments (**right**), including scale bars (50 µm) (** *p* < 0.01; *** *p* < 0.001; **** *p* < 0.0001).

WPMY-1 cells were exposed to different amounts of APOPROSTAT^®^ forte (0.3, 3, and 30 µg/mL) and to equivalent amounts of ethanol in controls. APOPROSTAT^®^ forte reduced the proliferation rate in WPMY-1 cells in a concentration-dependent and time-dependent manner (Figure 5A–C). An overview of the results can be seen in Supplementary File S8. After exposure to APOPROSTAT^®^ forte for 24 h, the proliferation rate was reduced to 53.3 [49.5 to 57.2]%, 45.2 [42.2 to 48.1]%, and 38.8 [34.8 to 42.7]% by 0.3, 3, and 30 µg/mL APOPROSTAT^®^ forte, respectively, while 68.7 [65.7 to 71.7]% of solvent-treated cells (ethanol) showed proliferation (*p* = 0.0016, *p* < 0.0001, and *p* = 0.0005 for 0.3, 3, and 30 µg/mL APOPROSTAT^®^ forte vs. control, respectively; Figure 5A). After exposure to APOPROSTAT^®^ forte for 48 h, the proliferation rate was reduced to 51.3 [47.9 to 54.8]%, 37.3 [30.1 to 43.8]%, and 31.2 [25.7 to 36.7]% by 0.3, 3, and 30 µg/mL APOPROSTAT^®^ forte, respectively, while 65.3 [62.3 to 68.4]% of solvent-treated cells (ethanol) showed proliferation (*p* = 0.0022, *p* = 0.0021, and *p* = 0.0006 for 0.3, 3, and 30 µg/mL APOPROSTAT^®^ forte vs. control, respectively; Figure 5B). After exposure to APOPROSTAT^®^ forte for 72 h, the proliferation rate was reduced to 44.2 [42.6 to 45.8]%, 30.1 [26.8 to 33.4]%, and 21.5 [17.9 to 25.1]% by 0.3, 3, and 30 µg/mL APOPROSTAT^®^ forte, respectively, while 59.8 [57.3 to 62.3]% of solvent-treated cells (ethanol) showed proliferation (*p* = 0.0003, *p* = 0.0001, and *p* < 0.0001 for 0.3, 3, and 30 µg/mL APOPROSTAT^®^ forte vs. control, respectively; Figure 5C). Relative inhibition of the proliferation rate was most pronounced at 64% with the highest concentration of APOPROSTAT^®^ forte after incubation for 72 h (Figure 5C).

#### 2.2.3. Effects of APOPROSTAT^®^ Forte on Colony Formation of WPMY-1 Cells

**Figure 6:**
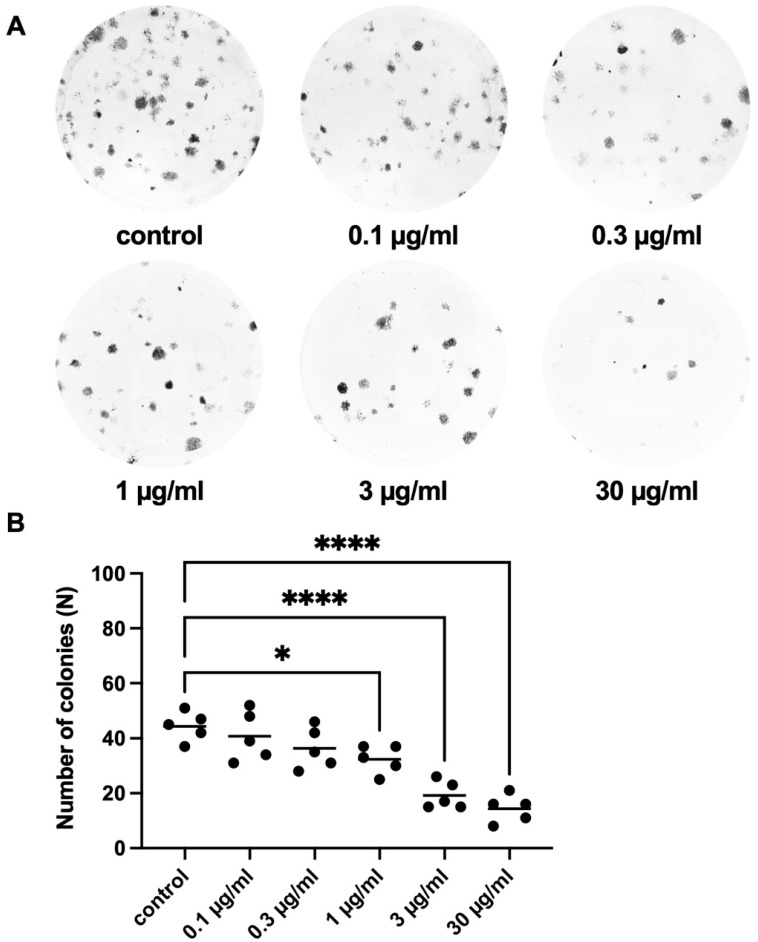
Effects of APOPROSTAT^®^ forte on colony formation of prostate stromal cells. Shown are the absolute numbers of colonies (n) after 168 h (single values with means) from series using cell cultures from *n* = 5 independent experiments. The cells were exposed to APOPROSTAT^®^ forte at 0.1, 0.3, 1, 3, or 30 μg/mL, or equal amounts of solvent (ethanol) for controls, and incubated for 168 h. Shown are exemplary images of colony formation after 168 h (**A**) and quantification of all experiments (**B**) (* *p* < 0.05; **** *p* < 0.0001).

Colony formation of WPMY-1 cells was assessed using different concentrations of APOPROSTAT^®^ forte, from 0.1 to 30 µg/mL (Figure 6A). APOPROSTAT^®^ forte significantly reduced colony formation only at concentrations of 1–30 µg/mL after 168 h (Figure 6B). The number of individual colonies per well amounted to 32 [26 to 39], 19 [13 to 25], and 14 [8 to 21] for 1, 3, and 30 µg/mL APOPROSTAT^®^ forte, respectively, compared to 44 [38 to 51] colonies for solvent-treated controls (*p* = 0.0259 and *p* < 0.0001 for 1, 3, and 30 µg/mL APOPROSTAT^®^ forte, respectively; Figure 6A). Therefore, the decline in colony formation was concentration-dependent and obviously reached a maximum at the highest applied concentration of 30 µg/mL APOPROSTAT^®^ forte, amounting to a relative reduction of individual cell colonies of about 68%, and an overview of results for each concentration can be found in Supplementary File S9.

#### 2.2.4. Effects of APOPROSTAT^®^ Forte on Viability of WPMY-1 Cells

**Figure 7:**
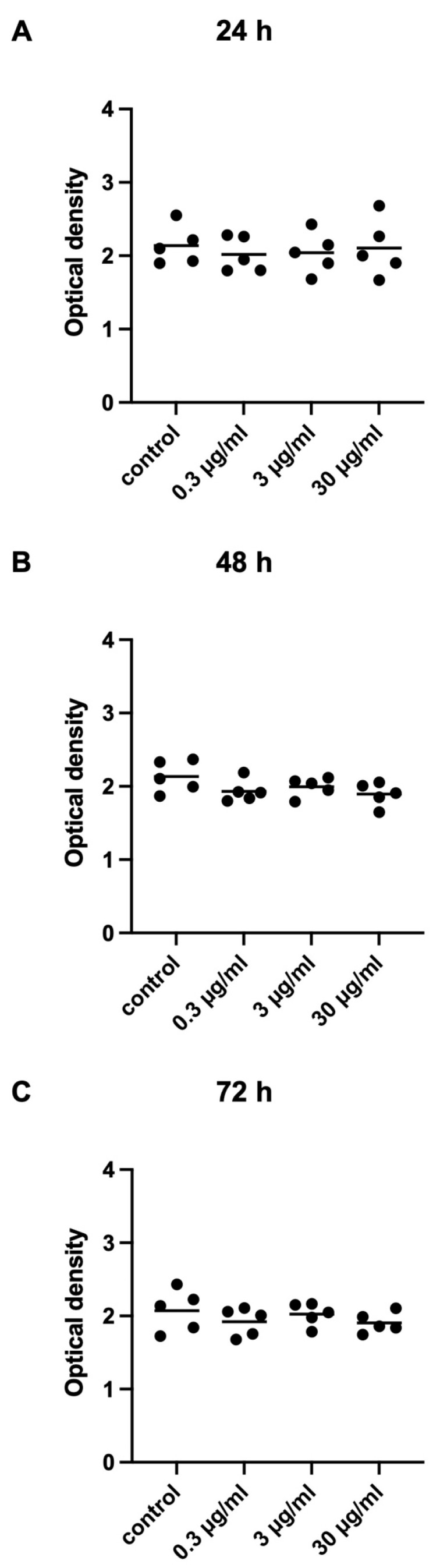
Effects of APOPROSTAT^®^ forte on viability of prostate stromal cells. To assess viability, a CCK-8 assay was used, and values are graphed as optical density (OD) from *n* = 5 individual experiments for each concentration and time. The cells were exposed to APOPROSTAT^®^ forte at 0.3, 3, or 30 μg/mL, or equal amounts of solvent (ethanol) for controls, and incubated for 24, 48, and 72 h (**A**–**C**). Shown are the quantifications of all experiments as optical density (mean ± SD).

Effects of APOPROSTAT^®^ forte (0.3–30 µg/mL) on viability of WPMY-1 cells was assessed in CCK-8 assays (Figure 7). Viability of WPMY-1 cells is depicted as optical density (OD), with values for each single experiment given in scatterplots. APOPROSTAT^®^ forte did not reduce viability of WPMY-1 cells at any concentration or time (Supplementary File S9).

#### 2.2.5. Effects of APOPROSTAT^®^ Forte on Apoptosis and Cell Death of WPMY-1 Cells

**Figure 8:**
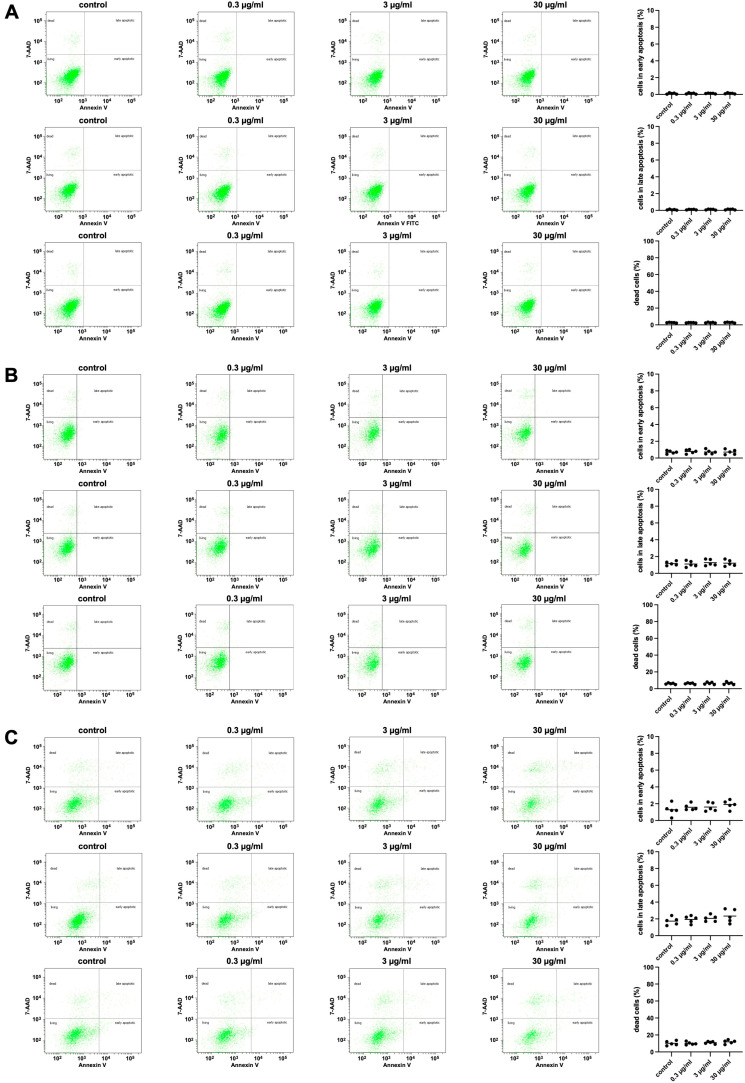
Effects of APOPROSTAT^®^ forte on apoptosis and cell death prostate stromal cells. WPMY-1 cells were incubated with different concentrations of APOPROSTAT^®^ forte, i.e., 0.3, 3, or 30 μg/mL, or equal amounts of solvent (ethanol) for controls. Flow cytometry was performed after cells were allocated to a control (ethanol) or drug (APOPROSTAT^®^ forte) group for 24 h (**A**), 48 h (**B**), or 72 h (**C**). Subsequently, the numbers of cells in early apoptosis (annexin V-positive, 7-AAD-negative) and late apoptosis (annexin V-positive, 7-AAD-positive) and those of dead cells (annexin V-negative, 7-AAD-positive; resulting from apoptosis and/or necrosis) were assessed by flow cytometry. Shown are single values from each experiment together with means (percentage of cells in early or late apoptosis or percentage of dead cells, relative to the number of all cells) and representative single experiments from a series of *n* = 5 independent experiments for each concentration and time.

Effects of APOPROSTAT^®^ forte (0.3–30 µg/mL) on apoptosis and cell death in WPMY-1 cells were assessed by flowcytometry analysis for annexin V and 7-AAD, where annexin V-positive/7-AAD-negative cells represent cells in early apoptosis (Figure 8), annexin V-positive/7-AAD-positive cells represent cells in late apoptosis, and annexin V-negative/7-AAD-positive cells represent dead cells. Following exposure of 24, 48, and 72 h, even at the highest concentration of 30 µg/mL, APOPROSTAT^®^ forte did not increase the relative numbers of cells in apoptosis or dead cells compared to ethanol-treated controls (Supplementary File S11).

## 3. Discussion

Although numerous clinical studies were conducted during the 1990s and early 2000s, the exact mechanisms of action of many phytopharmaceuticals remain unclear at the in vitro level. The following discussion is based on available data from the current body of literature. Additionally, we contextualized our study results on β-sitosterol (APOPROSTAT^®^ forte) within the existing body of literature and provided a comparative analysis to HESr. Following the 2021 update of the EAU guidelines on the management of non-neurogenic male LUTS and BPO, a formal recommendation for the use of phytotherapeutic agents was introduced—marking the first-ever endorsement of a plant extract by these urological guidelines: HESr [2]. Prior clinical trials have demonstrated that plant extracts improved male LUTS significantly better than placebo and comparable to α_1_-blockers [16]. With distinct differences in manufacturing processes, plant-based preparations are difficult to compare [30,31]. However, in addition to free fatty acids and lectins, phytosterols appear to be widely utilized active components in phytomedicines approved for LUTS/BPH [23,24,25]. Nonetheless, underlying mechanisms remain poorly understood and largely unexplored, marking an obvious discrepancy between patients’ demand of phytomedicines and their representation in high-quality randomized controlled trials (RCTs) [17,31]. Phytomedicines provide a promising complementary or alternative option for patients who want to avoid the bothersome side effects of synthetic medications for LUTS/BPH. Among these, β-sitosterol, a phytosterol structurally similar to cholesterol, shows clinical effectiveness in improving urinary symptoms with minimal adverse effects [32]. Although most studies focus on sources like saw palmetto and African plum bark, β-sitosterol is also present in the maritime pine bark (*Pinus pinaster*), which itself is rich in bioactive compounds such as procyanidins and phytosterols [33]. In vitro studies have shown that plant sterols from Pinus pinaster possess anti-inflammatory and antioxidant effects that may be highly relevant in BPH, where chronic inflammation and oxidative stress contribute to disease progression [26,34]. While β-sitosterol is also found in nuts, seeds, fruits, and plant oils, the bark extract of *Pinus pinaster* has been utilized as a source for urological use in APOPROSTAT^®^ forte. With a concentration of >70% β-sitosterol, APOPROSTAT^®^ forte has the highest phytosterol content currently available on the German market for LUTS/BPH treatment. While the in vitro research in the past primarily focused on the anti-inflammatory properties of β-sitosterol, the findings of our current study reveal a much more comprehensive picture of the vast positive properties of phytosterolic extracts for LUTS/BPH. Here, we show that β-sitosterol induces a concentration-dependent inhibition of smooth muscle contractions in both intact human prostate tissues and prostate stromal cells (WPMY-1), while simultaneously reducing the growth rate of prostate stromal cells and actin polymerization, without showing cytotoxic effects. These actions target key pathological processes involved in the development of male LUTS and align with the mechanisms addressed by current medical treatment options. Notably, this study provides for the first time new mechanistic insights for the clinical effects of phytosterol preparations, following the admission studies of highly concentrated β-sitosterol for the use in LUTS/BPH [27,28,29].

Smooth muscle contractility is one of the two main targets in pharmacotherapy for LUTS/BPH, as exaggerated prostate smooth muscle contraction contributes to BPO and obstructive voiding symptoms, i.e., weakened urinary stream, buildup of post-void residual, or incomplete emptying of the urinary bladder post-micturition [4,10]. It has become increasingly obvious that, in addition to α_1_-adrenoceptors, other so-called non-adrenergic receptors are also involved in the lower urinary tract as mediators of smooth muscle contraction. Not only do these mediators (thromboxane and endothelins) target their respective specific receptors, they are not inhibited by α_1_-blockers and may even complete contractions, which may explain the incomplete symptom relief with α_1_-blocker monotherapy [35].

While β-sitosterol is currently utilized for its potential health benefits, including the reduction of total cholesterol levels and the associated risk of cardiovascular disease when incorporated into a balanced diet [31], experimental animal studies have demonstrated the positive effects of β-sitosterol on BPH, highlighting its anti-androgenic, pro-apoptotic, anti-inflammatory, and antioxidant properties. However, despite these encouraging preclinical findings, translating these effects to human populations remains a significant challenge [36,37].

At concentrations ranging from 3 to 30 µg/mL, we observed inhibition of adrenergic prostate smooth muscle contraction of 25 to 63%, which increased with amounts of applied APOPROSTAT^®^ forte solution. While inhibition of α_1_-adrenergic smooth contraction is a mainstay in LUTS/BPH pharmacotherapy, our data are in the range of our previous data, in which an inhibition of adrenergic prostate contractions by tamsulosin (300 nM) was around 70% [35]. However, the focus of this study was to assess the effect of tamsulosin on non-adrenergic contractions, which could not be inhibited by the α_1_-blocker. Thus, following the observation of inhibitory effects of β-sitosterol on adrenergic prostate smooth muscle contraction, we further explored the non-adrenergic mediators, thromboxane A2 analog U46619 and endothelin-1. We observed significant and concentration-dependent inhibition of thromboxane A2- and endothelin-1-mediated prostate smooth muscle contraction by approximately 33 to 62% and 31 to 45%, respectively. These data not only underscore the potential of plant-based preparations for LUTS/BPH but also indicate that phytosterols such as β-sitosterol may be the main active ingredient. Our previous data regarding the effects of Permixon^®^ (HESr) showed limited efficacy regarding U46619-induced contractions and no effects on endothelin 1-induced contractions in human prostate and detrusor tissues [24]. Thus, β-sitosterol as an active ingredient may be responsible for the widely positive effects of phytomedicines, advocated for by phytosterol-enriched versions of saw palmetto [31,38]. Neurogenic contractions of smooth muscle tissues induced by EFS result from the activation of neuronal action potentials, followed by the release of endogenous neurotransmitters (primarily noradrenaline in prostate tissues), which subsequently activate postsynaptic α_1_-adrenoceptors [39]. We observed that APOPROSTAT^®^ forte produced a concentration-dependent inhibition of neurogenic contractions, achieving up to 55% reduction at the highest tested concentration. This degree of AUC inhibition falls well within the inhibitory range previously reported for HESr, where Permixon^®^ showed inhibition of around 60%, and for α_1_-blockers, where tamsulosin achieved 48–76% inhibition and silodosin achieved 47% inhibition of EFS-induced contractions under comparable experimental conditions using human prostate tissues in our previous studies [24,39,40,41].

While our data show clear and reproducible inhibition of adrenergic prostate smooth muscle contraction, involving intact tissue strips, the current body of literature explores the effects of β-sitosterol, ranging from antagonist of the muscarinic acetylcholine receptor subtype 3 (M3) [42] to attenuating vasorelaxation by increasing reactive oxygen species and cyclooxygenase-2 [43], or acting as a uterine stimulant, acting to inhibit potassium channels and sarcoplasmic reticulum calcium ATPase and thereby increasing contraction via calcium entry on L-type calcium channels and myosin light chain kinase [44]. Taken together, this highlights a wide variety of potential effects involving β-sitosterol and smooth muscle contraction.

In line with these data and by using a wide array of contractile agonists on human prostate tissues, our data point to anti-contractile activity of β-sitosterol in the human prostate. The observed inhibitory effects of APOPROSTAT^®^ forte on smooth muscle contraction may be either due to non-competitive receptor antagonism, as previously suggested [42], or by any other kind of inhibition of downstream post-receptor intracellular signaling, involving oxidation products [43]. The identification of active compound(s) and their specific molecular targets was beyond the scope of our study. Rather than comparing different plant-derived β-sitosterol extracts, we focused exclusively on the readily available OTC formulation of APOPROSTAT^®^ forte, which contains highly concentrated extracts of β-sitosterol from maritime pine bark. It remains plausible that either a single active constituent within the extract is responsible for all observed effects in both organ bath and cell culture experiments or multiple compounds contributed to the diverse effects detected. Although APOPROSTAT^®^ forte clearly reduced actin polymerization in our cell culture studies, this mechanism cannot account for the inhibition of smooth muscle contractions observed in the organ bath experiments. In these experiments, APOPROSTAT^®^ forte was applied for 24–72 h to cell cultures, but only 30 min to intact tissues, so that an actin breakdown may not necessarily account for the effects seen in organ bath.

Given that proper organization and polymerization of actin filaments are essential for smooth muscle contraction and that contractions in cell contraction assays were recorded up to 72 h, the observed decrease in actin polymerization could plausibly contribute to the reduction in contractility noted in the floating matrix assay. Indeed, the concentration-dependent reduction of AUC by up to 64% in the cell contraction assays aligns well with the pronounced reduction in phalloidin-stained areas, showing a 79% decrease in actin-stained areas at the highest concentration of APOPROSTAT^®^ forte and reflecting profound cytoskeletal remodeling. However, considering emerging studies demonstrating that β-sitosterol can inhibit proliferation and promote apoptosis in pulmonary arterial smooth muscle cells [45], it is likely that additional pathways beyond cytoskeletal remodeling are involved. Moreover, the effects observed in WPMY-1 prostate stromal cells might be mediated through alternative mechanisms, as we did not detect an increase in apoptosis rates in these cells.

The clinical efficacy of β-sitosterol-based phytomedicines for LUTS/BPH was proven in multiple clinical studies [27,28,29,46]. These studies demonstrated significant improvement of LUTS, after treatment with 20 mg β-sitosterol for three times per day for 3 to 6 months [29]. To cite specific pertinent results, patients reported significant improvement of nocturia with a reduction of Δ1.5 (1.7 SD ± 0.90) voids/night, an increase in Q_max_ of Δ4.5 ml/s (8.9 SD ± 8.86), and a decrease in IPSS by Δ5.4 points (8.2 SD ± 5.74), marking the clinically significant improvement, perceptible by the patient of ≥3 points [47]. Furthermore, improvement of symptoms was maintained at the 18-month follow-up [46]. Additionally, to put these data into perspective, the EAU’s guideline recommendation of HESr is based on the results of two systematic reviews of 12 and 15 RCTs [15,16]. Compared with placebo, HESr was associated with a reduction of nocturia by 0.64 voids/night, an additional mean increase in Q_max_ of 2.75 mL/s, and a mean significant improvement in IPSS of 5.73 points from baseline [16]. Notably, representative values for α_1_-blockers ranged around increases in Q_max_ of approximately 0.7–2.5 mL/s and decreases in IPSS of 3.8–6.6 points, in seminal clinical trials [48,49].

Furthermore, phytomedicines for LUTS/BPH are known for their superior tolerability and low rate of side effects [16,29]. A recent study on comparing tolerability and efficacy of HESr with tamsulosin reported only 2.1% adverse events (AEs) in patients receiving HESr, compared to 14.7% in the tamsulosin group [50]. AEs for phytomedicines are rare, mostly gastrointestinal, and amount to around 3.8% [16,29]. However, an explanation for these side effects may be through the inhibitory potential of β-sitosterol on gastrointestinal smooth muscle contraction. Recent findings by Aguirre-Crespo and colleagues suggest that triterpenes from *Coccoloba uvifera* act as a ligand of G protein-coupled receptors (GPCRs) and that β-sitosterol could act as an antagonist of muscarinic acetylcholine receptor subtype 3 (m3AChR) [42]. In the context of aqueous extracts of *C. uvifera*, β-sitosterol and their heterosides were identified by FTIR and ^1^H-NMR spectroscopy. The concerted binding of β-sitosterol and other triterpenes within the m3AChR binding site may be indicative of relaxant effects at the gastrointestinal smooth muscle level and explain TEAEs, such as diarrhea. Overall, clinical trials and observational studies suggest high efficacy, combined with excellent tolerability [17,46]. While multiple clinical trials have emerged since in vitro effects of β-sitosterol-based phytomedicines became known in the 1990s and early 2000s, the exact mechanisms of action of many phytopharmaceuticals remain unclear at the in vitro level and do not translate in vivo, or vice versa [27,28,29,46].

Several studies have attributed a 5α-reductase inhibitor (5-ARI)-like mechanism of action to HESr, based on a dose-dependent inhibition of intracellular DHT binding to cytosolic and nuclear receptors by liposterolic extracts of *Serenoa repens* in cultured human foreskin cells [51]. As noted above, the major phytosterol component of Permixon^®^ is β-sitosterol [25]. However, when assessing the effects of Permixon^®^ at calculated therapeutic concentrations on the activity of 5-AR isoenzymes, it was found to act as an effective inhibitor of both isoforms, without affecting the secretion of prostate specific antigen (PSA) from epithelial cells, even following stimulation with testosterone [52]. Electron microscopy revealed damage to the intracellular nuclear and mitochondrial membranes in epithelial cells and fibroblasts treated with Permixon^®^, which was not observed in untreated controls. Since 5-AR activity in the human prostate depends on the enzyme’s association with the nuclear membrane, disruption of the nuclear membrane, as observed in this study, could lead to inactivation of both 5-ARs [52,53,54]. Nevertheless, membrane damage induced by HESr is still considered the principal cause of inhibition of membrane-bound enzymatic activity. While the use of conventional 5-ARIs inevitably leads to a dramatic reduction in PSA expression by approximately 50%, Bayne and colleagues concluded that Permixon^®^ acts as a 5-ARI and further emphasized that PSA expression should not be equated with 5-AR activity [52,55]. Thus, it may be possible to achieve 5-AR inhibition without a corresponding decrease in PSA levels [52].

Comparable findings have also been reported for β-sitosterol, particularly in the context of androgenetic alopecia [56] and its effects on rodent prostates [57]. Notably, the results of the latter study by Cabeza and colleagues demonstrated that β-sitosterol, administered at various doses, significantly reduced the weight of testosterone-treated, castrated hamster prostates. β-sitosterol was also shown to inhibit the conversion of testosterone to DHT, exhibiting an IC_50_ value of 2.7 μM. Thus, β-sitosterol inhibited the activity of 5-AR present in hamster prostate tissue without displaying affinity for the androgen receptor in competitive binding assays. Consequently, Cabeza and colleagues concluded that the observed reduction in prostate weight was attributable to inhibition of 5-AR, rather than direct androgen receptor antagonism. Both mechanisms represent key therapeutic targets in the pharmacological management of LUTS/BPH. Such antiproliferative properties are frequently attributed to plant-based preparations. However, the results concerning β-sitosterol remain inconsistent. Some evidence suggests that β-sitosterol can inhibit proliferation in tumor cells, particularly in prostate, breast, colon, gastric, and lung carcinomas, as well as in leukemias [58,59]. Conversely, other studies indicate that phytosterols and pure β-sitosterol exert only minimal antiproliferative effects on malignant cell lines and show no significant antiproliferative effects on benign cell lines [60]. In our study, no impact on the viability of immortalized prostate stromal cells was observed. Specifically, no reduction in the survival of WPMY-1 cells was detected in the CCK-8 assay at any given time or concentration, following incubation with APOPROSTAT^®^ forte.

In our study with APOPROSTAT^®^ forte, a significant concentration- and time-dependent inhibition of prostate cell proliferation was observed. At the highest tested concentration of APOPROSTAT^®^ forte, an inhibition of approximately two-thirds in cell proliferation rate was recorded after 72 h of incubation. Even at lower concentrations, a statistically significant—albeit less pronounced—effect was still detectable. Considering the results regarding cell viability and cytotoxicity, the observed pronounced inhibition of proliferation appears to represent a true antiproliferative effect, rather than secondary to pro-apoptotic or cytotoxic mechanisms.

However, when Kassen and colleagues investigated the effects of β-sitosterol on transforming growth factor (TGF)-β1 expression in human prostate stromal cells, they found no impact on stromal cell proliferation rates when cells were cultured in the presence of β-sitosterol [61]. Regardless of the concentration used, β-sitosterol did not affect stromal cell proliferation. Consequently, Kassen and colleagues focused on examining the effects of β-sitosterol on the production of stromal cell-derived secretory proteins. In the presence of high concentrations of β-sitosterol, stromal cells exhibited an increased production and secretion of the multifunctional growth factor TGF-β1. The elevated TGF-β1 expression in β-sitosterol-treated cultures was markedly different from that observed in untreated controls or cholesterol-treated cultures. TGF-β1, as a multifunctional growth factor, may promote or inhibit growth activities, depending on its concentration and the target cell type [62]. Given that TGF-β1 can inhibit the growth of prostate epithelial cells, it is plausible that β-sitosterol might indirectly affect epithelial cell proliferation via stromal cell-derived TGF-β1. However, there is no evidence that β-sitosterol induces apoptosis in prostate cells, as the overall size of the prostate gland remains unchanged following β-sitosterol treatment [29,46]. Reflecting on the in vivo data by Berges and colleagues, the study concludes that another mechanism, rather than induction of apoptosis, must be responsible for the beneficial effects of sitosterol on BPH symptoms. A β-sitosterol-induced, long-term cytostatic effect of TGF-β1 on stromal and/or epithelial cells could represent a plausible explanation or potential mechanism for the positive clinical outcomes observed in men with LUTS/BPH in vivo and is in line with our findings. Prostate growth can be driven by various hormones and growth factors, with an androgen-regulated balance between inhibitory TGF-β and mitogenic cytokines like epidermal growth factor (EGF) and basic fibroblast growth factor (bFGF) playing a key role [63,64]. Age-related androgen decline may increase TGF-β expression, sensitizing prostate stem cells to proliferative signals and promoting hyperplasia, while EGF and bFGF can raise prostate cell proliferation by 30–200% [65,66]. While Permixon^®^ dose-dependently inhibits the bFGF- and EGF-induced proliferation of human prostate specimens [66], corresponding data for phytosterols are not yet available and may be the subject of follow-up studies. Nevertheless, and corresponding to our previous results regarding Permixon^®^ [24], we observed a concentration-dependent reduction in the number of colony-forming WPMY-1 cells in cell colony assay using APOPROSTAT^®^ forte.

These results present novel data facilitating a mechanistic link to the clinical efficacy of β-sitosterols in LUTS/BPH. Specifically, we demonstrate that APOPROSTAT^®^ forte exerts simultaneous inhibitory effects on agonist-induced and neurogenic smooth muscle contraction, stromal cell proliferation, and actin cytoskeletal organization, i.e., three critical processes involved in the pathophysiology of BPH. Importantly, these effects occur without inducing cytotoxicity or reducing cell viability, highlighting a targeted, non-lethal mode of action. In addition, our findings suggest that β-sitosterol may influence stromal and epithelial cell behavior indirectly through modulation of growth factors such as TGF-β1, contributing to a cytostatic environment rather than inducing apoptosis. Together, these results provide a plausible mechanistic explanation for the observed clinical improvements in LUTS symptoms with β-sitosterol-containing formulations and underscore the potential of APOPROSTAT^®^ forte as a multi-targeted therapeutic approach in the management of LUTS/BPH.

Our study has several limitations. Although it is possible that additional bioactive components were present in the APOPROSTAT^®^ forte extract, we consider it unlikely that the broad range of observed effects can be attributed substantially to compounds other than the highly concentrated phytosterol fraction (>70% β-sitosterol), which we regard as the principal active ingredient. Nevertheless, variations in the concentrations of bioactive constituents—even between batches from the same preparation—pose challenges for standardization and complicate direct comparisons of extract potency across different studies or production lots. Additionally, our findings were derived from in vitro and ex vivo experiments, and thus, their translation to in vivo outcomes remains uncertain. While the concentrations we used are well in line with achievable in vivo concentrations, we also explored concentrations well above and below the in vivo serum concentration in humans. This is not uncommon in experimental research and has in fact been previously demonstrated in experimental research. While APOPROSTAT^®^ forte is administered in vivo over a longer period of time, i.e., months up to years, we have exposed tissue strips in an organ bath for only 30 min incubation time. However, we would like to note that such experimental data may not directly be extrapolated to in vivo efficacy, and vice versa, as appropriate scaling algorithms in experimental research remain up for debate [67]. Additionally, in line with the experimental nature of our study, nonlinear regression was performed without predefined curve constraints and without reporting formal goodness-of-fit metrics. While this approach was chosen to avoid introducing bias into datasets with substantial biological variability and to maintain an exploratory analytical framework, it also means that statistical comparisons rely on two-way ANOVA. Instead, model plausibility and consistency were ensured by visual inspection of fitted curves for each experiment. Consequently, as in other studies using similar data structures, all *p*-values must be interpreted descriptively, as explicitly stated. However, our study is the first to demonstrate an inhibitory effect of β-sitosterol on human prostate smooth muscle contraction, supported by both tissue-based organ bath and cell culture models. These findings build upon prior research suggesting effects of β-sitosterol on smooth muscle cells and contractility [42,45], while also offering a novel mechanistic explanation for the consistently reported clinical benefits by demonstrating efficacy across multiple contractile agonists relevant to the lower urinary tract. Nonetheless, despite corroborating previous evidence for the biological activity of β-sitosterol, our conclusions are specifically limited to the APOPROSTAT^®^ forte formulation.

## 4. Materials and Methods

### 4.1. Materials, Drugs, and Nomenclature

APOPROSTAT^®^ forte (65 mg capsules) were provided by Apogepha Arzneimittel GmbH (Dresden, Germany). APOPROSTAT^®^ forte contains 65 mg phytosterols per capsule (>70% β-sitosterol, ethanol-extracted from maritime pine bark, *Pinus pinaster*), while additional components are refined peanut oil, pumpkin seed oil, refined rapeseed oil, all-rac-α-tocopherol (vitamin E) as an antioxidant, gelatin, glycerol, hard fat, polysorbate 80, de-oiled phospholipids from soybeans, iron (III) oxide (E 172), iron oxides and hydroxides (E 172), titanium dioxide (E 171), according to the manufacturer. Noradrenaline (4-[(1R)-2-amino-1-hydroxyethyl]-1,2-benzenediol), phenylephrine ((R)-3-[-1-hydroxy-2-(methylamino)ethyl] phenol), and methoxamine (α-(1-aminoethyl)-2,5-dimeth-oxy-benzyl alcohol) are agonists for α_1_-adrenoceptors. U46619 ((Z)-7-[(1-S,4R,5R,6S)-5-[(E,3S)-3-hydroxyoct-1-enyl]-3-oxabicyclo [2.2.1] heptan-6-yl]hept-5-enoic acid) is an analogue of thromboxane A_2_ and frequently used as an agonist for thromboxane receptors. Endothelin-1 is a 21-amino acid peptide with high affinity to the endothelin A (ET_A_) and B (ET_B_) receptors. Aqueous stock solutions of noradrenaline, phenylephrine, and methoxamine were freshly prepared before each experiment. Stock solutions of U46619 were prepared in ethanol and stock solutions of endothelin-1 in water, and both were stored at −80 °C until use. Noradrenaline, phenylephrine, and methoxamine were obtained from Sigma (Munich, Germany), and U46619 and endothelin-1 were obtained from Enzo Life Sciences (Lörrach, Germany).

### 4.2. Preparation of APOPROSTAT^®^ Forte

In clinical practice, APOPROSTAT^®^ forte 65 mg capsules are administered twice daily (once in the morning and once in the evening), totaling to 130 mg/day [27]. Assuming distribution in total body fluid achievable using the recommended therapeutic dosage, a man with average weight of 78 kg consists of 55% body fluid. Thus, dissolving the daily dose of 130 mg APOPROSTAT^®^ forte in 42.9 L water yields 3.03 mg/L, which is equivalent to ~3 µg/mL. We dissolved the contents of one capsule of APOPROSTAT^®^ forte 65 mg with 6.5 mL ethanol. For this purpose, one capsule was opened, and the contents were resuspended in 6.5 mL ethanol, followed by incubation in a water bath (37 °C) for two hours. Afterwards, the tube was centrifuged at 500× *g* for 3 min, and the supernatant was aliquoted into working solutions with a concentration designed to achieve volume equivalent amounts of solvent across various final concentrations. For example, a 30 µL stock solution was taken, amounting to a total of 0.3 mg APOPROSTAT^®^ forte, and applied to an organ bath chamber containing 10 mL Krebs−Henseleit solution, resulting in a final concentration of 0.03 mg/mL (i.e., 30 µg/mL). For cell culture experiments, the stock solution was further diluted to a concentration of 0.1 mg/mL APOPROSTAT^®^ forte. From this, the same procedure was repeated for all cell culture experiments. For example, a 1 µL stock solution, amounting to a total of 0.001 mg APOPROSTAT^®^ forte, was applied to 10 mL FCS-free medium to achieve a final concentration of 0.0001 mg/mL (i.e., 0.1 µg/mL). This dilution process was repeated with 3, 10, 30, and 300 µL of stock solution. For controls, volume equivalent amounts of ethanol were used. Thus, working solutions were prepared for APOPROSTAT^®^ forte in organ baths and cell culture experiments, so that final concentrations resulted in 0.99% for ethanol in each organ bath chamber or cell culture well.

### 4.3. Organ Bath

For organ bath experiments, human prostate tissues were obtained from patients who underwent radical prostatectomy for prostate cancer (*n* = 100) at our tertiary referral center. Our research was carried out in accordance with the Declaration of Helsinki of the World Medical Association and has been approved by the ethics committee of Ludwig-Maximilians University, Munich, Germany. Informed consent was obtained from all patients. All samples and data were collected and analyzed anonymously. Tissue sampling and subsequent tension measurements were performed as described previously for prostate specimens [68,69,70]. In addition to generating concentration−response curves, EC_50_ values for contractile agonists, Ef_50_ values for frequency-induced contractions (f), and E_max_ values were determined through curve fitting. Curve fitting and calculation of the area under curves (AUC) was conducted individually for each experiment using GraphPad Prism version 9.3.0 (GraphPad Software Inc., San Diego, CA, USA), and the resulting values were analyzed as outlined below. Curve fitting was performed separately for each experiment, resulting in distinct values for each independent dataset. Both frequency−response and concentration−response curves were fitted using nonlinear regression without applying predefined constraints for minimum (bottom), maximum (top), or EC_50_ values. An ordinary fit method was used, with no weighting and no automatic outlier exclusion. The resulting parameter values were reviewed for plausibility. If error messages occurred during curve fitting, settings were adjusted in accordance with recommendations from the “GraphPad Curve Fitting Guide” (GraphPad Software Inc., San Diego, CA, USA).

### 4.4. Cell Culture

Cell culture experiments were conducted using the immortalized human prostate stromal cell line WPMY-1, derived from nonmalignant tissue, as previously described [68]. The cells were obtained from the American Type Culture Collection (ATCC; Manassas, VA, USA) and cultured in RPMI 1640 medium (Gibco, Carlsbad, CA, USA) supplemented with 10% fetal calf serum (FCS) and 1% penicillin/streptomycin. Cultures were maintained at 37 °C in a humidified atmosphere with 5% CO_2_. Prior to the addition of APOPROSTAT^®^ forte or ethanol, the culture medium was replaced with serum-free medium. The medium was subsequently refreshed daily until cells reached confluence. Following cell counting and volume adjustment based on experimental requirements, cells were seeded into appropriate culture vessels for subsequent experiments.

#### 4.4.1. Cell Contraction Assay

The contractility of WPMY-1 cells was assessed using the Floating Matrix Model of the CytoSelect™ 24-Well Cell Contraction Assay Kit (Cell Biolabs, San Diego, CA, USA), as previously described [71]. Cells were cultured in 75 cm^2^ flasks for 48 h. After incubation, cells were trypsinized and resuspended in fresh RPMI medium at a concentration of 10^7^ cells/mL and then transferred to 24-well assay plates for contraction analysis. APOPROSTAT^®^ forte at concentrations of 0.3, 3, and 30 µg/mL or an equivalent volume of ethanol (used as a control) was added 24 h prior to trypsinization. Each well was filled with a matrix mixture composed of 100 μL of the trypsinized cell suspension and 400 μL of the collagen gel working solution provided in the kit. This mixture was thoroughly combined before being dispensed into the wells. The plates were then incubated for 1 h at 37 °C with 5% CO_2_ to allow for collagen polymerization. Following this, 1 mL of RPMI medium, containing either APOPROSTAT^®^ forte or the ethanol control, was added to each well, and incubation continued under the same conditions. To monitor collagen contraction, images were captured at 1, 2, 3, 6, 12, 24, 48, and 72 h after RPMI addition. Using ImageJ software (version 1.53c), the diameter of the collagen plugs and wells were measured in the images. Contraction was calculated as the difference between the well diameter and the plug diameter at each time point and expressed in millimeters (mm).

#### 4.4.2. Phalloidin Staining

For fluorescence staining with phalloidin, WPMY-1 cells were seeded at a density of 30,000 cells per well on 16-well chambered coverslips and cultured for 24 h. Following this initial incubation, cells were treated with predetermined concentrations of APOPROSTAT^®^ forte, while control wells received equivalent volumes of ethanol. Treatments were continued for an additional 24, 48, and 72 h. Staining of filamentous actin (F-actin) was performed using 100 μM fluorescein isothiocyanate (FITC)-conjugated phalloidin (cat. no. P5282, Sigma-Aldrich, St. Louis, MO, USA), following the manufacturer’s protocol. To visualize cell nuclei, counterstaining was carried out with DAPI (4′,6-diamidino-2-phenylindole). Fluorescently labeled cells were then examined using a laser scanning confocal microscope (Leica SP8 AOBS WLL, Wetzlar, Germany) and analyzed as previously described [68,69].

#### 4.4.3. Cell Proliferation Assay

Proliferation of WPMY-1 cells was assessed by “EdU-Click 555” cell proliferation assay (Baseclick, Tutzing, Germany), as previously described [68,69]. Confluent cells were treated with APOPROSTAT^®^ forte or ethanol and grown for 24, 48, or 72 h. The number of proliferating cells (i.e., EdU-stained cells) was calculated as percentage of all cells individually for each sample, as previously described [70]. Thus, EdU incorporation into DNA was visualized using fluorescence labeling with 5-carboxytetramethylrhodamine (5-TAMRA). To counterstain all nuclei, DAPI (4′,6-diamidino-2-phenylindole; cat. no. D1306, Invitrogen by Fisher Scientific GmbH, Schwerte, Germany) was applied. Fluorescence microscopy was performed to analyze the samples (excitation: 546 nm; emission: 479 nm), and 25 representative images were captured per well. Blinding was employed as a method of randomization. Each experiment was independently repeated five times, with each coverslip representing a separate experiment. The number of proliferating cells, identified as EdU-positive, was quantified within the microscopic fields using the ImageJ Cell Counter tool (U.S. National Institutes of Health, Bethesda, MD, USA). For each experiment, the proliferation rate was calculated by dividing the number of EdU-positive cells by the total number of DAPI-stained nuclei, yielding a proliferation ratio.

#### 4.4.4. Plate Colony Assay

Colony formation of WPMY-1 cells was assessed as previously described [68,69]. WPMY-1 cells were seeded at a density of 100 cells per well in Falcon 6-well tissue culture plates and incubated at 37 °C for 168 h. Following this initial growth phase, confluent cells were exposed either to APOPROSTAT^®^ forte or to ethanol for 168 h, and the number of cell colonies was counted individually for each sample. Subsequently, cells were washed twice with phosphate-buffered saline (PBS) and fixed overnight at 4 °C using 2 mL of 10% trichloroacetic acid. Plates were then washed five times with cold water and stained with a 0.4% sulforhodamine B (Sigma-Aldrich, St. Louis, Missouri, USA) solution prepared in 1% acetic acid for 30 min at room temperature. Prior to analysis, plates were labeled and rinsed five times with 1% acetic acid to remove unbound dye. Colony formation was assessed under white light, and the number of cell colonies was quantified for each individual experiment using the ImageJ Cell Counter.

#### 4.4.5. Cell Viability Assay

The effect of APOPROSTAT^®^ forte on cell viability was assessed using the Cell Counting Kit-8 (CCK-8) (Sigma-Aldrich, St. Louis, MO, USA), as previously described [68,69]. WPMY-1 cells were seeded in 96-well plates at a density of 20,000 cells per well and cultured for 24 h. Following this incubation period, cells were treated with predetermined concentrations of APOPROSTAT^®^ forte, while control wells received equivalent volumes of ethanol. For each concentration and time point, a series of five independent experiments (*n* = 5) was conducted. Cells were then incubated with the respective concentrations of APOPROSTAT^®^ forte for 24, 48, or 72 h, with separate control groups. At the end of each incubation period, 10 µL of WST-8 reagent (2-(2-methoxy-4-nitrophenyl)-3-(4-nitrophenyl)-5-(2,4-disulfophenyl)-2H-tetrazolium monosodium salt) from the CCK-8 kit was added to each well. After 120 min of incubation at 37 °C, the optical density (OD) was measured at 450 nm to determine cell viability.

#### 4.4.6. Flow Cytometry Analysis for Apoptosis and Cell Death

A flow cytometry-based apoptosis detection kit utilizing annexin V allophycocyanin (APC) and 7-aminoactinomycin D (7-AAD) (BD Biosciences, Franklin Lakes, NJ, USA) was employed to identify apoptotic cells (annexin V-positive, 7-AAD-negative) and dead cells (annexin V-positive, 7-AAD-positive). WPMY-1 cells were seeded in Falcon 6-well tissue culture plates at a density of 250,000 cells per well and incubated at 37 °C for 24 h. Following this, cells were treated with APOPROSTAT^®^ forte at defined concentrations, while control wells received equal volumes of ethanol. Cells were then incubated for an additional 24, 48, and 72 h. After treatment, cells were washed with PBS and resuspended in annexin V binding buffer (cat. no. 556454, BD Biosciences). Each sample was then stained with 5 µL APC-conjugated annexin V (cat. no. 550475, BD Biosciences) and 5 µL of 7-AAD solution (cat. no. 559925, BD Biosciences). Samples were incubated for 15 min at room temperature in the dark, followed by the addition of 400 µL binding buffer prior to analysis. Flow cytometric analysis was carried out using a BD FACSCalibur™ Cytometer (BD Biosciences), with data processed using FloJo software v8.8.7 (FlowJo LLC, Ashland, Oregon). Gating strategies were optimized using fluorescence-minus-one (FMO) controls, which involved excluding one fluorochrome at a time from the staining panel to correctly define gates, along with additional controls for untreated/unstained, treated/unstained, and untreated/stained cells [72]. Initially, forward scatter (FSC) and side scatter (SSC) gating were used to identify viable cells and exclude debris and aggregates. WPMY-1 cells were distinguished from cell fragments based on their position in FSC-A vs. SSC-A plots, with smaller debris appearing near the origin. Singlets were then isolated by plotting FSC-A (area) against FSC-H (height), allowing exclusion of doublets or cell clumps, which typically exhibit a disproportionately high area relative to height. To minimize spectral overlap, background signals from non-target fluorochromes were assessed using appropriate compensation settings. APC-annexin V was excited using a 635 nm red laser, while 7-AAD was excited with a 488 nm blue laser. For each sample, 10,000 events were recorded and analyzed using ModFit LT™ software (Version 2)(Verity Software House, Topsham, ME, USA). This approach enabled precise quantification of apoptotic and dead cells following exposure to the respective compounds.

### 4.5. Data and Statistical Analysis

Data from frequency and concentration−response curves are presented as mean values ± standard deviation (SD) in graphical format. Parameters such as E_max_, pEC_50_, and Ef_50_, and results from cell culture experiments are shown as individual data points from each independent experiment. These are displayed alongside the respective means in scatter plots, with matching symbols used to indicate related samples from the same experiment. Effect sizes are illustrated in the frequency− and concentration−response graphs and scatter plots, and are summarized in simplified form within the main text. A detailed overview, including comparisons between the effects of APOPROSTAT^®^ forte and control tissues, is provided in Supplementary File S1. Only statistically significant values for E_max_, pEC_50_, and Ef_50_ are explicitly stated in the text.

Statistical analysis was conducted using GraphPad Prism, version 9.3.0 (GraphPad Software Inc., San Diego, CA, USA). Comparisons of entire frequency and concentration-response curves were performed using two-way analysis of variance (ANOVA), while post hoc analyses at each concentration in concentration−response curves were performed using Sidak’s multiple comparisons. For each experiment, the area under the curve (AUC) was calculated for frequency− and concentration−response curves under both treatment (APOPROSTAT^®^ forte) and control (ethanol) conditions. AUC values, along with their 95% confidence intervals, are reported for each frequency, concentration, and experimental series. AUCs are reported in arbitrary units (a.u.) due to their dimensionless nature. E_max_, pEC_50_, and Ef_50_ values were derived for each experiment via curve fitting and reported with the corresponding absolute mean differences (MDs). Curve fitting was carried out as described above using GraphPad Prism 9.3.0. Comparisons of E_max_, pEC_50_, and Ef_50_ values between groups were conducted using paired Student’s *t*-tests. The study followed an exploratory design, and statistical testing was not intended to evaluate a pre-defined null hypothesis, as discussed previously [73]. As such, all *p*-values are reported descriptively and should not be interpreted as confirmatory. A minimum of five experiments per group (*n* = 5) was pre-specified to enable meaningful statistical comparisons. Once five or more replicates were completed for a given condition, and data were analyzed. If initial findings suggested a potential effect without reaching statistical significance (*p* ≥ 0.05), additional experiments were performed, and the data reanalyzed. This flexible sampling strategy is permissible within the context of exploratory studies and is appropriate when dealing with biological variability [74]. Importantly, interim analyses were limited to descriptive plotting of frequency− and concentration−response curves and did not include curve fitting, which was reserved for the completion of full data sets. No data or experiments were excluded from the analysis.

## 5. Conclusions

In summary, our study provides the first direct evidence that APOPROSTAT^®^ forte, an OTC phytotherapeutic formulation with highly concentrated phytosterol (>70% β-sitosterol) derived from maritime pine bark (*Pinus pinaster*), exerts a concentration-dependent inhibitory effect on human prostate smooth muscle contraction, both in organ bath experiments and in cell culture models. Simultaneously, APOPROSTAT^®^ forte inhibited prostate stromal cell proliferation and actin cytoskeletal polymerization, without showing cytotoxic effects, inducing apoptosis, or compromising cell viability. Thus, our data underscore the significance of phytosterols as a main bioactive component in many phytomedicines, strengthening the mechanistic foundation underlying the proven clinical efficacy of β-sitosterol-containing formulations. By simultaneously targeting smooth muscle contraction and stromal cell proliferation, the main components of BPH pathophysiology, β-sitosterol likely addresses multiple key drivers of LUTS development, supporting the promising therapeutic potential of APOPROSTAT^®^ forte in the management of male LUTS.

## Data Availability

The original contributions presented in this study are included in the article. Further inquiries can be directed to the corresponding author.

## Supplementary Materials

The following supporting information can be downloaded at: https://www.mdpi.com/article/10.3390/ph18121864/s1.

## Abbreviations

5-AR	5α-reductase
5-ARI	5α-reductase inhibitor
7-AAD	7-aminoactinomycin D
APC	allophycocyanin
ATCC	American Type Culture Collection
BPH	benign prostatic hyperplasia
CCK-8	cell counting kit 8
DHT	dihydrotestosterone
EAU	European Association of Urology
EdU	5-ethynyl-2′-deoxyuridine
ETA	endothelin receptor A
ETB	endothelin receptor B
FCS	fetal calf serum
HeSr	hexane-extracted Serenoa repens
LUTS	lower urinary tract symptoms
OAB	overactive bladder
OD	optical density
OTC	over the counter
RCT	randomized controlled trial
RPMI	Roswell Park Memorial Institute
WPMY-1	immortalized human prostate stromal cell line (benign)
