# Supplementary material for: Pine-Extracted Phytosterol β-Sitosterol (APOPROSTAT® Forte) Inhibits Both Human Prostate Smooth Muscle Contraction and Prostate Stromal Cell Growth, Without Cytotoxic Effects: A Mechanistic Link to Clinical Efficacy in LUTS/BPH
Source: Pharmaceuticals (Basel). 2025 Dec 6;18(12):1864. doi: 10.3390/ph18121864 (PMC12735939; doi:10.3390/ph18121864)
Supplement: Supplementary file 1 [file pharmaceuticals-18-01864-s001.zip › pharmaceuticals-3966160-supplementary.pdf]

Supplementary file 1

(A) AUC	30 µg/ml		10 µg/ml		3 µg/ml	
MD [95 % CI]	**Apoprostat®**	**control**	**Apoprostat®**	**control**	**Apoprostat®**	**control**
**NA**	388 [260 to 515]	866 [671 to 1060]	558 [372 to 745]	844 [582 to 1105]	530 [426 to 634]	749 [594 to 905]
**PE**	234 [140 to 329]	631 [491 to 771]	434 [271 to 597]	696 [516 to 876]	573 [380 to 765]	764 [598 to 931]
**MXE**	316 [230 to 402]	689 [522 to 855]	361 [245 to 478]	576 [478 to 674]	446 [310 to 581]	605 [496 to 714]
**U46619**	117 [78 to 155]	305 [228 to 382]	122 [84 to 160]	240 [160 to 319]	136 [64 to 209]	202 [134 to 270]
**ET-1**	327 [158 to 496]	597 [342 to 852]	352 [261 to 443]	545 [334 to 757]	358 [258 to 459]	520 [381 to 679]
**EFS**	115 [48 to 182]	253 [185 to 320]	110 [71 to 149]	230 [164 to 296]	118 [54 to 182]	180 [95 to 264]

(B) Emax	30 µg/ml		10 µg/ml		3 µg/ml	
	**Apoprostat®**	**control**	**Apoprostat®**	**control**	**Apoprostat®**	**control**
**NA**	92 [33 to 151]	240 [114 to 366]	159 [88 to 230]	208 [105 to 312]	141 [101 to 182]	201 [124 to 277]
**PE**	154 [70 to 238]	216 [155 to 276]	150 [76 to 223]	226 [162 to 290]	212 [102 to 322]	233 [177 to 289]
**MXE**	146 [99 to 192]	218 [154 to 283]	158 [96 to 220]	228 [155 to 302]	192 [97 to 286]	246 [208 to 284]
**U46619**	80 [27 to 133]	158 [22 to 293]	96 [25 to 168]	117 [70 to 163]	87 [45 to 130]	134 [97 to 170]
**ET-1**	131 [43 to 218]	240 [116 to 364]	171 [116 to 226]	153 [153 to 352]	161 [118 to 203]	273 [181 to 366]
**EFS**	55 [23 to 87]	101 [72 to 130]	61 [35 to 87]	90 [70 to 110]	87 [40 to 133]	121 [39 to 202]

(C) pEC50/Ef50	30 µg/ml		10 µg/ml		3 µg/ml	
	**Apoprostat®**	**control**	**Apoprostat®**	**control**	**Apoprostat®**	**control**
**NA**	6.5 [5.6 to 7.4]	5.7 [5.0 to 6.4]	5.8 [5.2 to 6.4]	6.1 [6.0 to 6.3]	5.8 [5.6 to 6.1]	5.9 [5.4 to 6.5]
**PE**	4.5 [3.1 to 5.8]	5.4 [4.9 to 5.8]	5.4 [5.1 to 5.7]	5.5 [4.9 to 6.0]	5.4 [4.9 to 6.0]	5.6 [5.3 to 5.9]
**MXE**	5.1 [4.7 to 5.5]	5.4 [5.0 to 5.9]	5.2 [4.8 to 5.6]	5.3 [4.8 to 5.7]	5.1 [4.9 to 5.3]	5.2 [4.9 to 5.5]
**U46619**	5.2 [4.7 to 5.4]	5.5 [4.6 to 6.4]	5.5 [4.6 to 6.5]	5.6 [4.8 to 6.4]	5.0 [4.5 to 5.5]	5.0 [4.6 to 5.4]
**ET-1**	6.4 [6.2 to 6.5]	6.4 [5.8 to 6.9]	6.1 [6.0 to 6.3]	6.2 [5.9 to 6.4]	6.3 [6.0 to 6.6]	6.0 [5.7 to 6.3]
**EFS**	11.9 [4.7 to 19.1]	13.1 [7.2 to 18.3]	12.9 [7.6 to 18.3]	8.8 [3.2 to 14.4]	14.5 [10.0 to 19.0]	14.3 [10.0 to 18.7]

Effects of APOPROSTAT® forte on adrenergic. non-adrenergic. and neurogenic prostate contraction (detailed results). Presented are Area Under the Curve (AUC) (A), Emax (B), and pEC50/Ef50 values (C) for concentration-response curves following incubation with APOPROSTAT® forte (at 30, 10, and 3 µl/ml) and equivalent amounts of ethanol (control). (A) shows the AUC values with 95% confidence interval (CI), (B) shows the Emax values with 95% CI expressed as a percentage (%), and (C) shows the pEC50 values for contractile agonists and Ef50 values for electrical field stimulation (EFS), each with 95% CI. The contractions were calculated and normalized to the contraction induced by high-molar KCl. MCH = Methacholine; CCH = Carbachol; U46619 = Thromboxane A2 analog; ET-1 = Endothelin-1; NA = Noradrenaline; PE = Phenylephrine; MXE = Methoxamine; EFS = Electrical Field Stimulation.

Supplementary file 2

	MD	95 % CI	p value	
**NA [µM]**				
**0.1**	22.92	-42.78 to 88.63	0.9337	ns
**0.3**	48.02	-17.68 to 113.7	0.2675	ns
**1**	29.43	-36.27 to 95.13	0.8003	ns
**3**	99.3	33.60 to 165.0	0.0011	**
**10**	101	35.34 to 166.7	0.0009	***
**30**	121.6	55.85 to 187.3	<0.0001	****
**100**	135	69.28 to 200.7	<0.0001	****
**PE [µM]**				
**0.1**	17.37	-48.76 to 83.49	0.9855	ns
**0.3**	19.38	-46.74 to 85.50	0.9732	ns
**1**	51.05	-15.07 to 117.2	0.2131	ns
**3**	69.55	3.432 to 135.7	0.0348	*
**10**	95.91	29.79 to 162.0	0.0017	**
**30**	106.5	40.38 to 172.6	0.0005	***
**100**	91.77	25.65 to 157.9	0.0028	**
**MXE [µM]**				
**0.1**	14.72	-45.82 to 75.26	0.9914	ns
**0.3**	33.03	-27.50 to 93.57	0.6198	ns
**1**	57.55	-2.993 to 118.1	0.0706	ns
**3**	78.27	17.73 to 138.8	0.0054	**
**10**	76.27	15.73 to 136.8	0.007	**
**30**	79.67	19.13 to 140.2	0.0045	**
**100**	80.88	20.34 to 141.4	0.0038	**
**U46619 [µM]**				
**0.01**	11.6	-14.01 to 37.22	0.7504	ns
**0.03**	29.08	3.458 to 54.69	0.0201	*
**0.1**	41.76	16.15 to 67.38	0.0006	***
**0.3**	43.66	18.05 to 69.28	0.0003	***
**1**	39.56	13.94 to 65.18	0.0011	**
**3**	56.38	30.76 to 82.00	<0.0001	****
**ET-1 [µM]**				
**0.01**	12.11	-72.07 to 96.30	0.9973	ns
**0.03**	42.81	-41.38 to 127.0	0.6061	ns

**0.1**	77.16	-7.023 to 161.3	0.084	ns
**0.3**	91.65	7.467 to 175.8	0.0281	*
**1**	103.8	19.58 to 187.9	0.0106	*
**EFS [Hz]**				
**2**	30.99	2.474 to 59.51	0.029	*
**4**	33.25	4.731 to 61.77	0.0175	*
**8**	27.36	-1.160 to 55.88	0.0641	ns
**16**	35.38	6.857 to 63.90	0.0108	*
**32**	52.46	23.94 to 80.98	0.0002	***

Effects of APOPROSTAT® forte (30 µl/ml) on adrenergic, non-adrenergic, and neurogenic prostate contraction (detailed results). The results are presented as mean difference (MD) with 95% confidence interval (CI) for control group (ethanol) vs. APOPROSTAT® forte (30 µl/ml) for each individual concentration and each agonist used. Post hoc tests were performed using Šidák correction, and the resulting p-values are shown alongside the 95% CI. MCH = Methacholine; CCH = Carbachol; U46619 = Thromboxane A2 analog; ET-1 = Endothelin-1; EFS = Electrical Field Stimulation; µM = Micromolar; Hz = Hertz; ns = not significant; *p<0.05; **p<0.01; ***p<0.001; ****p<0.0001.

Supplementary file 3

	MD	95 % CI	p value	
**NA [µM]**				
**0.1**	5.99	-31.33 to 43.31	0.9994	ns
**0.3**	35.3	-2.019 to 72.62	0.0728	ns
**1**	44.3	6.978 to 81.62	0.0124	*
**3**	56.21	18.89 to 93.53	0.0009	***
**10**	63.49	26.17 to 100.8	0.0002	***
**30**	56.56	19.24 to 93.88	0.0009	***
**100**	52.69	15.37 to 90.01	0.002	**
**PE [µM]**				
**0.1**	6.139	-33.65 to 45.93	0.9995	ns
**0.3**	23.45	-16.34 to 63.24	0.5189	ns
**1**	32.67	-7.123 to 72.46	0.1601	ns
**3**	44.42	4.630 to 84.22	0.0219	*
**10**	55.47	15.68 to 95.27	0.0027	**
**30**	66.31	26.52 to 106.1	0.0003	***
**100**	73.9	34.11 to 113.7	<0.0001	****
**MXE [µM]**				
**0.1**	6.826	-36.70 to 50.36	0.9995	ns
**0.3**	11.73	-31.80 to 55.26	0.9844	ns
**1**	20.7	-22.83 to 64.23	0.7596	ns
**3**	39.49	-4.042 to 83.02	0.0945	ns
**10**	53.01	9.482 to 96.54	0.0098	**
**30**	54.9	11.37 to 98.43	0.0069	**
**100**	62.89	19.36 to 106.4	0.0016	**
**U46619 [µM]**				
**0.01**	3.988	-20.25 to 28.22	0.998	ns
**0.03**	9.524	-14.71 to 33.76	0.8575	ns
**0.1**	18.27	-5.963 to 42.51	0.2275	ns
**0.3**	26.37	2.130 to 50.60	0.0272	*
**1**	39.77	15.53 to 64.00	0.0004	***
**3**	43.39	19.15 to 67.62	0.0001	***
**ET-1 [µM]**				
**0.01**	12.54	-47.42 to 72.49	0.9847	ns
**0.03**	28.51	-31.44 to 88.46	0.6689	ns

**0.1**	50.89	-9.060 to 110.8	0.1255	ns
**0.3**	70	10.05 to 130.0	0.0165	*
**1**	75.98	16.02 to 135.9	0.0083	**
**EFS [Hz]**				
**2**	8.539	-14.55 to 31.63	0.84	ns
**4**	21.31	-1.781 to 44.41	0.0798	ns
**8**	39.15	16.06 to 62.24	0.0005	***
**16**	38.3	15.21 to 61.39	0.0007	***
**32**	33.55	10.46 to 56.65	0.0027	**

Effects of APOPROSTAT® forte (10 µl/ml) on adrenergic, non-adrenergic, and neurogenic prostate contraction (detailed results). The results are presented as mean difference (MD) with 95% confidence interval (CI) for control group (ethanol) vs. APOPROSTAT® forte (10 µl/ml) for each individual concentration and each agonist used. Post hoc tests were performed using Šidák correction, and the resulting p-values are shown alongside the 95% CI. MCH = Methacholine; CCH = Carbachol; U46619 = Thromboxane A2 analog; ET-1 = Endothelin-1; EFS = Electrical Field Stimulation; µM = Micromolar; Hz = Hertz; ns = not significant; *p<0.05; **p<0.01; ***p<0.001; ****p<0.0001.

Supplementary file 4

	MD	95 % CI	p value	
**NA [µM]**				
**0.1**	0.7674	-60.83 to 62.37	>0.9999	ns
**0.3**	6.919	-54.68 to 68.52	>0.9999	ns
**1**	28.8	-32.80 to 90.40	0.7651	ns
**3**	45.31	-16.29 to 106.9	0.2608	ns
**10**	48.15	-13.45 to 109.7	0.2018	ns
**30**	66.29	4.689 to 127.9	0.0293	*
**100**	46.34	-15.26 to 107.9	0.2381	ns
**PE [µM]**				
**0.1**	11.55	-42.10 to 65.20	0.9959	ns
**0.3**	20.15	-33.50 to 73.79	0.9109	ns
**1**	20.16	-33.48 to 73.81	0.9105	ns
**3**	40.9	-12.74 to 94.55	0.2303	ns
**10**	53.7	0.05499 to 107.4	0.0496	*
**30**	36.02	-17.63 to 89.67	0.3705	ns
**100**	30.5	-23.15 to 84.14	0.5725	ns
**MXE [µM]**				
**0.1**	3.34	-62.20 to 68.88	>0.9999	ns
**0.3**	6.1	-59.44 to 71.64	>0.9999	ns
**1**	20.42	-45.11 to 85.96	0.9651	ns
**3**	17.84	-47.70 to 83.38	0.9834	ns
**10**	35.56	-29.98 to 101.1	0.6263	ns
**30**	55.33	-10.21 to 120.9	0.1417	ns
**100**	45.31	-20.22 to 110.8	0.336	ns
**U46619 [µM]**				
**0.01**	3.413	-17.82 to 24.65	0.9983	ns
**0.03**	6.465	-14.77 to 27.70	0.9524	ns
**0.1**	6.47	-14.77 to 27.71	0.9523	ns
**0.3**	14.35	-6.886 to 35.59	0.339	ns
**1**	19.44	-1.799 to 40.67	0.0873	ns
**3**	34.55	13.32 to 55.79	0.0005	***
**ET-1 [µM]**				
**0.01**	10.83	-46.95 to 68.62	0.9906	ns

**0.03**	16.47	-41.32 to 74.25	0.9429	ns
**0.1**	15.28	-42.50 to 73.07	0.9579	ns
**0.3**	78.93	21.15 to 136.7	0.0042	**
**1**	90.88	33.09 to 148.7	0.001	***
**EFS [Hz]**				
**2**	7.378	-33.24 to 48.00	0.9912	ns
**4**	14.06	-26.56 to 54.68	0.8728	ns
**8**	3.054	-37.57 to 43.67	0.9999	ns
**16**	24.42	-16.20 to 65.04	0.4215	ns
**32**	31.98	-8.644 to 72.60	0.1725	ns

Effects of APOPROSTAT® forte (3 µl/ml) on adrenergic, non-adrenergic, and neurogenic prostate contraction (detailed results). The results are presented as mean difference (MD) with 95% confidence interval (CI) for control group (ethanol) vs. APOPROSTAT® forte (3 µl/ml) for each individual concentration and each agonist used. Post hoc tests were performed using Šidák correction, and the resulting p-values are shown alongside the 95% CI. MCH = Methacholine; CCH = Carbachol; U46619 = Thromboxane A2 analog; ET-1 = Endothelin-1; EFS = Electrical Field Stimulation; µM = Micromolar; Hz = Hertz; ns = not significant; *p<0.05; **p<0.01; ***p<0.001.

Supplementary file 5

**Cell Contraction**	**MD [95 % CI]**	**0.3 µg/ml**	**3 µg/ml**	**30 µg/ml**
**contraction (mm)**	**control**	**Apoprostat®**	**Apoprostat®**	**Apoprostat®**
**1 h**	2.0 [1.6 to 2.4]	1.9 [1.8 to 2.0]	1.0 [0.6 to 1.4]	0.4 [0.3 to 0.6]
**2 h**	2.6 [2.0 to 3.3]	2.4 [2.1 to 2.7]	1.3 [0.8 to 1.8]	0.8 [0.6 to 1.0]
**3 h**	3.7 [3.4 to 4.1]	3.0 [2.7 to 3.4]	1.7 [1.4 to 2.0]	1.1 [1.0 to 1.2]
**4 h**	4.1 [3.7 to 4.4]	3.1 [2.7 to 3.5]	1.8 [1.6 to 2.1]	1.3 [1.1 to 1.4]
**5 h**	4.6 [4.1 to 5.1]	3.2 [2.9 to 3.5]	2.0 [1.7 to 2.2]	1.4 [1.3 to 1.5]
**6 h**	5.3 [5.1 to 5.6]	3.6 [3.3 to 3.9]	2.6 [2.5 to 2.7]	2.0 [1.9 to 2.2]
**12 h**	5.8 [5.6 to 6.1]	3.7 [3.4 to 4.0]	2.7 [2.6 to 2.8]	2.1 [2.0 to 2.1]
**24 h**	6.3 [5.9 to 6.6]	3.8 [3.5 to 4.0]	2.9 [2.7 to 3.2]	2.5 [2.2 to 2.8]
**48 h**	6.6 [6.3 to 7.0]	3.8 [3.6 to 4.1]	3.3 [3.0 to 3.6]	2.7 [2.5 to 3.0]
**72 h**	6.8 [6.7 to 6.9]	3.9 [3.7 to 4.1]	3.4 [3.1 to 3.6]	2.8 [2.7 to 3.0]

Effects of APOPROSTAT® forte on contraction of WPMY-1 cells (detailed results). The results are presented as mean difference (MD) with 95% confidence interval (CI) for the control group (ethanol) vs. APOPROSTAT® forte (0.3, 3, and 30 µl/ml) at each time point. The MD refers to the absolute change in the maximum matrix plug diameter (15 mm).

Supplementary file 6

Time (h)	MD	95 % CI	p value	
**Comparisons**				
**1**				
**control vs. 0.3 µg/ml**	0.082	-0.4389 to 0.6029	>0.9999	ns
**control vs. 3 µg/ml**	0.982	0.4611 to 1.503	<0.0001	****
**control vs. 30 µg/ml**	1.522	1.001 to 2.043	<0.0001	****
**0.3 µg/ml vs. 3 µg/ml**	0.9	0.3791 to 1.421	<0.0001	****
**0.3 µg/ml vs. 30 µg/ml**	1.44	0.9191 to 1.961	<0.0001	****
**3 µg/ml vs. 30 µg/ml**	0.54	0.01909 to 1.061	0.033	*
**2**				
**control vs. 0.3 µg/ml**	0.228	-0.2929 to 0.7489	0.9999	ns
**control vs. 3 µg/ml**	1.35	0.8291 to 1.871	<0.0001	****
**control vs. 30 µg/ml**	1.834	1.313 to 2.355	<0.0001	****
**0.3 µg/ml vs. 3 µg/ml**	1.122	0.6011 to 1.643	<0.0001	****
**0.3 µg/ml vs. 30 µg/ml**	1.606	1.085 to 2.127	<0.0001	****
**3 µg/ml vs. 30 µg/ml**	0.484	-0.03691 to 1.005	0.107	ns
**3**				
**control vs. 0.3 µg/ml**	0.718	0.1971 to 1.239	0.0004	***
**control vs. 3 µg/ml**	2.01	1.489 to 2.531	<0.0001	****
**control vs. 30 µg/ml**	2.616	2.095 to 3.137	<0.0001	****
**0.3 µg/ml vs. 3 µg/ml**	1.292	0.7711 to 1.813	<0.0001	****
**0.3 µg/ml vs. 30 µg/ml**	1.898	1.377 to 2.419	<0.0001	****
**3 µg/ml vs. 30 µg/ml**	0.606	0.08509 to 1.127	0.0071	**
**4**				
**control vs. 0.3 µg/ml**	0.956	0.4351 to 1.477	<0.0001	****
**control vs. 3 µg/ml**	2.214	1.693 to 2.735	<0.0001	****
**control vs. 30 µg/ml**	2.798	2.277 to 3.319	<0.0001	****
**0.3 µg/ml vs. 3 µg/ml**	1.258	0.7371 to 1.779	<0.0001	****
**0.3 µg/ml vs. 30 µg/ml**	1.842	1.321 to 2.363	<0.0001	****
**3 µg/ml vs. 30 µg/ml**	0.584	0.06309 to 1.105	0.012	*
**5**				
**control vs. 0.3 µg/ml**	1.316	0.7951 to 1.837	<0.0001	****

**control vs. 3 µg/ml**	2.572	2.051 to 3.093	<0.0001	****
**control vs. 30 µg/ml**	3.17	2.649 to 3.691	<0.0001	****
**0.3 µg/ml vs. 3 µg/ml**	1.256	0.7351 to 1.777	<0.0001	****
**0.3 µg/ml vs. 30 µg/ml**	1.854	1.333 to 2.375	<0.0001	****
**3 µg/ml vs. 30 µg/ml**	0.598	0.07709 to 1.119	0.0086	**
**6**				
**control vs. 0.3 µg/ml**	1.698	1.177 to 2.219	<0.0001	****
**control vs. 3 µg/ml**	2.782	2.261 to 3.303	<0.0001	****
**control vs. 30 µg/ml**	3.32	2.799 to 3.841	<0.0001	****
**0.3 µg/ml vs. 3 µg/ml**	1.084	0.5631 to 1.605	<0.0001	****
**0.3 µg/ml vs. 30 µg/ml**	1.622	1.101 to 2.143	<0.0001	****
**3 µg/ml vs. 30 µg/ml**	0.538	0.01709 to 1.059	0.0345	*
**12**				
**control vs. 0.3 µg/ml**	2.15	1.629 to 2.671	<0.0001	****
**control vs. 3 µg/ml**	3.182	2.661 to 3.703	<0.0001	****
**control vs. 30 µg/ml**	3.77	3.249 to 4.291	<0.0001	****
**0.3 µg/ml vs. 3 µg/ml**	1.032	0.5111 to 1.553	<0.0001	****
**0.3 µg/ml vs. 30 µg/ml**	1.62	1.099 to 2.141	<0.0001	****
**3 µg/ml vs. 30 µg/ml**	0.588	0.06709 to 1.109	0.0109	*
**24**				
**control vs. 0.3 µg/ml**	2.504	1.983 to 3.025	<0.0001	****
**control vs. 3 µg/ml**	3.328	2.807 to 3.849	<0.0001	****
**control vs. 30 µg/ml**	3.74	3.219 to 4.261	<0.0001	****
**0.3 µg/ml vs. 3 µg/ml**	0.824	0.3031 to 1.345	<0.0001	****
**0.3 µg/ml vs. 30 µg/ml**	1.236	0.7151 to 1.757	<0.0001	****
**3 µg/ml vs. 30 µg/ml**	0.412	-0.1089 to 0.9329	0.3754	ns
**48**				
**control vs. 0.3 µg/ml**	2.784	2.263 to 3.305	<0.0001	****
**control vs. 3 µg/ml**	3.36	2.839 to 3.881	<0.0001	****
**control vs. 30 µg/ml**	3.904	3.383 to 4.425	<0.0001	****
**0.3 µg/ml vs. 3 µg/ml**	0.576	0.05509 to 1.097	0.0145	*
**0.3 µg/ml vs. 30 µg/ml**	1.12	0.5991 to 1.641	<0.0001	****
**3 µg/ml vs. 30 µg/ml**	0.544	0.02309 to 1.065	0.0302	*
**72**				

**control vs. 0.3 µg/ml**	2.848	2.327 to 3.369	<0.0001	****
**control vs. 3 µg/ml**	3.402	2.881 to 3.923	<0.0001	****
**control vs. 30 µg/ml**	3.954	3.433 to 4.475	<0.0001	****
**0.3 µg/ml vs. 3 µg/ml**	0.554	0.03309 to 1.075	0.0241	*
**0.3 µg/ml vs. 30 µg/ml**	1.106	0.5851 to 1.627	<0.0001	****
**3 µg/ml vs. 30 µg/ml**	0.552	0.03109 to 1.073	0.0252	*

Effects of APOPROSTAT® forte on contraction of WPMY-1 cells with multiple comparisons (detailed results). The results are presented as mean difference (MD) with 95% confidence interval (CI) for the control group (ethanol) vs. APOPROSTAT® forte (0.3, 3, and 30 µl/ml) at each time point. Multiple comparisons between individual groups were performed as post hoc tests using Šidák correction, and the resulting p-values are shown alongside the 95% CI (ns = not significant; *p<0.05; **p<0.01; ***p<0.001; ****p<0.0001).

Supplementary file 7

**Phalloidin Assay**		**0.3 µg/ml**	**3 µg/ml**	**30 µg/ml**
**Phalloidin-stained area (%)**	**control**	**Apoprostat®**	**Apoprostat®**	**Apoprostat®**
**24 h**	74.6 [72.9 to 76.3]	60.9 [55.4 to 66.5]	55.4 [51.5 to 59.2]	50.0 [46.7 to 53.2]
**48 h**	74.0 [69.9 to 78.0]	49.3 [46.1 to 52.6]	41.8 [35.3 to 48.3]	30.7 [25.3 to 36.0]
**72 h**	74.7 [70.1 to 79.3]	45.5 [41.3 to 49.7]	36.3 [30.8 to 41.9]	15.5 [11.2 to 19.9]

Effects of APOPROSTAT® forte on actin polymerization and organization of actin filaments in prostate stromal cells (detailed results). WPMY-1 cells were incubated with different concentrations of APOPROSTAT® forte (0.3, 3, or 30 µg/ml) or with equal amounts of solvent (ethanol) for the control group. Shown are the mean percentages of phalloidin-stained areas with corresponding 95% confidence intervals. for each concentration and time point (24 h, 48 h, and 72 h).

Supplementary file 8

EdU Assay	MD [95 % CI]	0.3 µg/ml	3 µg/ml	30 µg/ml
**Proliferation (%)**	**control**	**Apoprostat®**	**Apoprostat®**	**Apoprostat®**
**24 h**	68.7 [65.7 to 71.7]	53.3 [49.5 to 57.2]	45.2 [42.2 to 48.1]	38.8 [34.8 to 42.7]
**48 h**	65.3 [62.3 to 68.4]	51.3 [47.9 to 54.8]	37.3 [30.1 to 43.8]	31.2 [25.7 to 36.7]
**72 h**	59.8 [57.3 to 62.3]	44.2 [42.6 to 45.8]	30.1 [26.8 to 33.4]	21.5 [17.9 to 25.1]

Effects of APOPROSTAT® forte on proliferation of prostate stromal cells (detailed results). WPMY-1 cells were incubated with various concentrations of APOPROSTAT® forte (0.3, 3, or 30 µg/ml) or with equal amounts of solvent (ethanol) for the control groups. Shown are the mean percentages of proliferating cells with corresponding 95% confidence intervals. for each concentration and time point (24 h, 48 h, and 72 h).

Supplementary file 9

Cell Colony Assay		0.1 µg/ml	0.3 µg/ml
**Cell colonies (n)**	**control**	**Apoprostat®**	**Apoprostat®**
**168 h**	44 [38 to 51]	41 [30 to 52]	36 [27 t0 46]

	**1 µg/ml**	**3 µg/ml**	**30 µg/ml**
	**Apoprostat®**	**Apoprostat®**	**Apoprostat®**
	32 [26 to 39]	19 [13 to 25]	14 [8 to 21]

Effects of APOPROSTAT® forte on colony formation of prostate stromal cells (detailed results). Shown are the mean values (mean) as the absolute number of cell colonies (n) after 168 hours. with corresponding 95% confidence intervals. for each concentration (0.1, 0.3, 1, 3, and 30 µl/ml APOPROSTAT® forte).

Supplementary file 10

CCK-8 Assay		0.3 µg/ml	3 µg/ml	30 µg/ml
**Optical Density (OD)**	**control**	**Apoprostat®**	**Apoprostat®**	**Apoprostat®**
**24 h**	2.1 [1.8 to 2.5]	2.0 [1.7 to 2.3]	2.0 [1.7 to 2.4]	2.1 [1.6 to 2.6]
**48 h**	2.1 [1.9 to 2.4]	1.9 [1.7 to 2.1]	2.0 [1.8 to 2.1]	1.9 [1.7 to 2.1]
**72 h**	2.1 [1.7 to 2.4]	1.9 [1.7 to 2.2]	2.0 [1.8 to 2.2]	1.9 [1.7 to 2.1]

Effects of APOPROSTAT® forte on the viability of prostate stromal cells (detailed results). WPMY-1 cells were incubated with various concentrations of APOPROSTAT® forte (0.3, 3, or 30 µg/ml) or with equal amounts of solvent (ethanol) for the control groups. Shown are the mean values (mean) as optical density (OD) with corresponding 95% confidence intervals. for each concentration and time point (24 h, 48 h, and 72 h).

Supplementary file 11

FACS		0.3 µg/ml	3 µg/ml	30 µg/ml
**(I) early apoptosis (%)**		**Apoprostat®**	**Apoprostat®**	**Apoprostat®**
**24 h**	0.10 [0.01 to 0.20]	0.11 [0.02 to 0.19]	0.11 [0.06 to 0.16]	0.12 [0.04 to 0.19]
**48 h**	0.70 [0.47 to 0.93]	0.78 [0.49 to 1.06]	0.73 [0.40 to 1.05]	0.72 [0.35 to 1.08]
**72 h**	1.28 [0.40 to 2.16]	1.58 [1.10 to 2.06]	1.60 [0.96 to 2.24]	1.90 [1.25 to 2.55]
**(II) late apoptosis (%)**				
**24 h**	0.08 [0.01 to 0.14]	0.09 [0.04 to 0.14]	0.10 [0.03 to 0.16]	0.11 [0.04 to 0.17]
**48 h**	1.17 [0.87 to 1.47]	1.11 [0.67 to 1.55]	1.27 [0.80 to 1.74]	1.22 [0.75 to 1.68]
**72 h**	1.74 [1.16 to 2.32]	1.94 [1.40 to 2.49]	2.06 [1.59 to 2.53]	2.54 [1.18 to 3.91]
**(III) dead cells (%)**				
**24 h**	2.70 [2.28 to 3.11]	2.64 [2.31 to 2.98]	2.68 [2.12 to 3.25]	2.84 [2.32 to 3.36]
**48 h**	5.98 [4.88 to 7.08]	6.14 [5.11 to 7.18]	6.43 [4.74 to 8.11]	6.35 [4.52 to 8.17]
**72 h**	9.40 [7.16 to 11.64]	10.06 [7.90 to 12.22]	11.98 [9.20 to 14.76]	14.84 [11.56 to 18.12]

Effects of APOPROSTAT® forte on apoptosis and cell death in prostate stromal cells (detailed results). WPMY-1 cells were incubated with various concentrations of APOPROSTAT® forte (0.3, 3, or 30 µg/ml) or with equal amounts of solvent (ethanol) for the control groups. Shown are the mean values (mean) of the relative number of cells in early apoptosis (Annexin V-positive, 7-AAD-negative [A]), late apoptosis (Annexin V-positive, 7-AAD-positive [B]), and dead cells (Annexin V-negative, 7-AAD-positive [C]; resulting from apoptosis and/or necrosis), for each concentration and time point (24 h, 48 h, and 72 h).
